# Imaging Markers of Post-Stroke Depression and Apathy: a Systematic Review and Meta-Analysis

**DOI:** 10.1007/s11065-017-9356-2

**Published:** 2017-08-22

**Authors:** Elles Douven, Sebastian Köhler, Maria M. F. Rodriguez, Julie Staals, Frans R. J. Verhey, Pauline Aalten

**Affiliations:** 1 Department of Psychiatry and Neuropsychology, School for Mental Health and Neuroscience (MHeNS), Alzheimer Center Limburg, Maastricht University, Dr. Tanslaan 12, PO Box 616 (DRT 12), 6200 MD Maastricht, The Netherlands; 2 Hospital Alvaro Cunqueiro, Department of Psychiatry, Complexo Universitario de Vigo, Vigo, Spain; 3 Department of Neurology, Cardiovascular Research Institute Maastricht (CARIM), Maastricht University Medical Center, Maastricht, The Netherlands

**Keywords:** Stroke, Depression, Apathy, Imaging, Systematic review, Meta-analysis

## Abstract

Several brain imaging markers have been studied in the development of post-stroke depression (PSD) and post-stroke apathy (PSA), but inconsistent associations have been reported. This systematic review and meta-analysis aims to provide a comprehensive and up-to-date evaluation of imaging markers associated with PSD and PSA. Databases (Medline, Embase, PsycINFO, CINAHL, and Cochrane Database of Systematic Reviews) were searched from inception to July 21, 2016. Observational studies describing imaging markers of PSD and PSA were included. Pooled odds ratios (OR) and 95% confidence intervals (CI) were calculated to examine the association between PSD or PSA and stroke lesion laterality, type, and location, also stratified by study phase (acute, post-acute, chronic). Other imaging markers were reviewed qualitatively. The search retrieved 4502 studies, of which 149 studies were included in the review and 86 studies in the meta-analyses. PSD in the post-acute stroke phase was significantly associated with frontal (OR 1.72, 95% CI 1.34–2.19) and basal ganglia lesions (OR 2.25, 95% CI 1.33–3.84). Hemorrhagic stroke related to higher odds for PSA in the acute phase (OR 2.58, 95% CI 1.18–5.65), whereas ischemic stroke related to higher odds for PSA in the post-acute phase (OR 0.20, 95% CI 0.06–0.69). Frequency of PSD and PSA is modestly associated with stroke type and location and is dependent on stroke phase. These findings have to be taken into consideration for stroke rehabilitation programs, as this could prevent stroke patients from developing PSD and PSA, resulting in better clinical outcome.

**Electronic supplementary material:**

The online version of this article (doi:10.1007/s11065-017-9356-2) contains supplementary material, which is available to authorized users.

## Introduction

Post-stroke depression (PSD) and post-stroke apathy (PSA) are frequent neuropsychiatric symptoms after stroke, with estimated prevalence rates between 30 and 40%, respectively, in the first few months after stroke (Hackett et al. 2014). Depression can be defined as a feeling of low mood, loss of interest, and lack of pleasure that persists for a time period of at least 2 weeks (Hackett et al. 2005). Apathy is generally defined as a disorder of diminished motivation, characterized by loss of interest, diminished emotional response, and loss of initiative (Marin 1990), and can occur independently (Levy et al. 1998), or in combination with symptoms of depression (Marin et al. 1993).

According to a previous meta-analysis, approximately 40% of patients with PSA also suffer from PSD (van Dalen et al. 2013). Because depression and apathy share several features, mainly loss of interest, patients with apathy after stroke are frequently misdiagnosed as having PSD (Hama et al. 2011). However, despite the considerable overlap in symptoms between PSD and PSA, there is evidence indicating that the two syndromes seem to develop from different anatomical and neurobiological constructs (Andersson et al. 1999; Hama et al. 2007b, 2016; Hollocks et al. 2015; Murakami et al. 2013).

Earlier studies have already attempted to disentangle the relationship between PSD and PSA, which have shown inconclusive results. The Sydney Stroke Study showed evidence for independence of PSA and PSD when measuring 3 to 6 months post-stroke (Brodaty et al. 2005). However, at 1-year follow-up, a significant overlap between apathy and depression was found (Withall et al. 2011). Contrastingly, Caeiro et al. (2013b) did not find an association between PSA and PSD at 1-year post-stroke. It is important to disentangle the relationship between PSA and PSD, at least from a clinical perspective, since the two syndromes seem to benefit from different types of medication (Withall et al. 2011). Both PSD and PSA are known to have a negative influence on clinical outcome (Hama et al. 2007a; Pohjasvaara et al. 2001) and quality of life (Carod-Artal et al. 2000; Mayo et al. 2009). Early treatment and prevention of PSD and PSA might have a positive effect on functional outcome, thereby limiting the impact of stroke on patients’ daily lives (Ramasubbu and Kennedy 1994). Identification of associated risk factors is thus important for early detection and tailoring of rehabilitation programs.

Several brain imaging markers have been studied in the development of PSD. Early studies suggested that PSD is frequent in patients with left frontal lesions (Brodaty et al. 2005; Hama et al. 2007b), but this hypothesis could not be supported by later studies (Carson et al. 2000). Previous systematic reviews primarily looked at the relationship with lesion laterality, while other potential imaging markers such as lesion location, lesion type, lesion volume, white matter hyperintensities (WMH), and atrophy have been ignored (Carson et al. 2000; Kutlubaev and Hackett 2014; Wei et al. 2015). In addition, imaging markers of PSA have been studied less frequently compared with PSD. Some studies provided evidence that PSA is associated with right hemispheric and subcortical lesions (Andersson et al. 1999; Caeiro et al. 2012; Starkstein et al. 1993), though two recent systematic reviews on lesion location in PSA reported inconclusive results (Caeiro et al. 2013a; van Dalen et al. 2013). Caeiro et al. (2013a) only studied the association with lesion laterality and were not able to find an association, whereas van Dalen et al. provided a qualitative overview of associations with lesion location and laterality, but no meta-analysis, and concluded that no clear association with lesion side or location could be found, though associations with the basal ganglia were most consistent.

A systematic review and meta-analysis was performed to evaluate the association between different brain imaging markers and PSD and PSA, thereby updating and extending previous meta-analyses just focusing on lesion location in association with PSD and PSA. The main aim of the present study was to investigate differences and similarities in several brain correlates associated with PSD and PSA.

## Methods

### Search Strategy and Selection Criteria

This systematic review and meta-analysis was conducted according to the Preferred Reporting Items for Systematic Reviews and Meta-Analyses (PRISMA) statement (Liberati et al. 2009) and by use of a predefined research protocol. Databases (Medline, Embase, PsycINFO, CINAHL, and Cochrane Database of Systematic Reviews) were searched from inception to December 2015 and updated to July 21, 2016. A full description of the search strategy is presented in supplementary Online Resource 1. To be eligible for inclusion, studies had to a) include patients with ischemic or hemorrhagic stroke, b) assess the presence of depressive or apathetic symptoms, c) examine the association between these symptoms and an imaging marker, d) the population had to be human adults, e) sample size had to be larger than 25 to avoid inclusion of spurious associations from underpowered studies, and f) language had to be English, German, Dutch, or French. Studies were excluded if (a) the study population was other than stroke or a combined population was studied without separate results available for stroke, (b) the population consisted of only patients with cognitive impairment or dementia in which vascular damage (infarcts, WMH, atrophy) was studied, or (c) no imaging data or lesion-related data (e.g. lesion location, type, laterality, WMH) were described. Records of research protocols, reviews, and abstracts from scientific meetings were excluded. If studies presented results from the same cohort on a certain outcome measure, data from the study that used the largest group of patients were used, or if they used an equal number of patients, data from the earliest publication were used.

Two reviewers (E.D. and P.A.) independently screened titles and abstracts manually for potential eligibility. Doubtful records were discussed (E.D. and P.A.) and an independent third reviewer (S.K.) decided if doubtful cases were included or not for full-text scrutiny. For completeness, reference lists were screened for additional articles. One reviewer (E.D.) assessed eligibility for inclusion based on full-text screening.

### Data Collection and Extraction

Data extraction was performed on selected articles for which full texts were obtained. Two independent reviewers (E.D. and M.R.) extracted data from each study according to a predefined data extraction form. The following information was extracted for each study included in the review: (1) first author and year of publication, (2) demographic characteristics, (3) in- and exclusion criteria if specified, (4) imaging method and imaging markers, (5) questionnaires and criteria used to define PSD or PSA, (6) time of measurement after stroke, (7) statistical methods used and results needed for the meta-analysis, (8) main conclusion and limitations.

### Statistical Analyses and Study Quality

Statistical analyses were performed using STATA 13.1 (StataCorp, TX, USA). Statistical significance was defined by *p* < .05 in two-sided tests. Pooled odds ratios (ORs) with corresponding 95% confidence intervals (CIs) were calculated to examine the association between PSD or PSA and stroke lesion laterality, type, and location, also stratified by study phase (acute, post-acute, chronic), using a DerSimonian-Laird random-effects model to account for within- and between-study variance (DerSimonian and Laird 1986). A full description of the observations used in the meta-analyses is presented in supplementary Online Resource 2. Studies were stratified according to phase in which they measured depression or apathy after stroke: acute (< 15 days from stroke onset), post-acute (15 days–6 months), and chronic phase (> 6 months). This stratification was based on the meta-analysis by Caeiro et al. (2013a) and was chosen because the acute stroke phase corresponds with the period of hospitalization and acute care (Buisman et al. 2015) and in this period the risk of complications and recurrent stroke is highest (Prasad et al. 2011). After this period of acute care, patients usually start with rehabilitation and most recovery will take place in the first 6 months (Aziz 2010). Therefore, we defined this period as the post-acute period, and > 6 months as the chronic stroke phase.

To identify possible sources of heterogeneity, random-effect meta-regression models were conducted including the following covariates: study phase, mean age, PSD/PSA prevalence, percentage of females, imaging method (CT/MRI vs. MRI), patient source, and first-ever stroke (yes/no).

The study quality was assessed by a single investigator (E.D.) with the Newcastle-Ottowa Scale (NOS) for case-control and cohort studies, and a modified NOS was applied for cross-sectional studies (Wells et al. 2000). A maximum score of 9 can be obtained and studies with a score < 5 were not included in the meta-analyses (see supplementary Online Resource 3). Visual inspection of asymmetry in funnel plots, in which the relation between sample size and effect size is assessed, was used to test for possible publication bias. Egger’s regression tests were performed to test for significant asymmetry of funnel plots as a test for small-study effects (Egger et al. 1997).

## Results

**Fig. 1:**
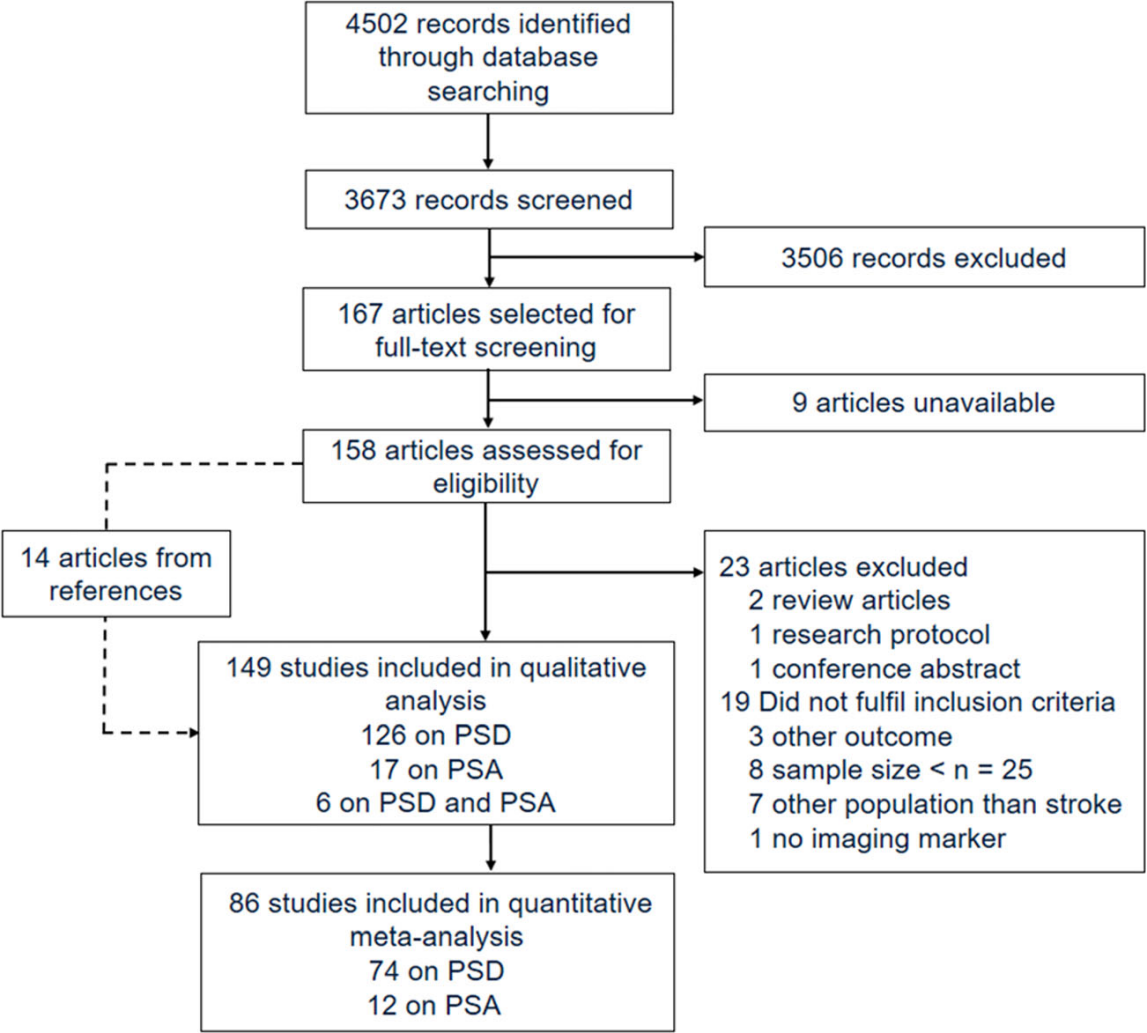
Preferred Reporting Items for Systematic Reviews and Meta-analyses (PRISMA) flowchart of study selection and review. *PSA* post-stroke apathy, *PSD* post-stroke depression

Of 4502 identified articles, 167 articles were selected for full-text screening (see Fig. 1). Nine articles could not be retrieved from authors after several contact requests. Based on full-text evaluation of the remaining articles, 135 articles met inclusion criteria. Reasons for exclusion were: conference abstract (Akiashvili et al. 2013), research protocol (Toso et al. 2004), review article (Beckson and Cummings 1991; Robinson and Starkstein 1989), small sample size (Beblo et al. 1999; Grasso et al. 1994; Lassalle-Lagadec et al. 2012, 2013; Matsuoka et al. 2015; Mayberg et al. 1988; Paradiso et al. 2013; Ramasubbu et al. 1999), other outcome than depression or apathy (Astrom 1996; Downhill and Robinson 1994; Vataja et al. 2005), study population other than stroke or no separate results available for stroke subpopulation (Bella et al. 2010; Grool et al. 2013; O’Brien et al. 2006; Ojagbemi et al. 2013; Sachdev et al. 2007; Tanislav et al. 2015; Wu et al. 2014), and no evaluation of imaging markers (Eriksen et al. 2016). Fourteen additional studies found in reference lists and fulfilling eligibility criteria were included, resulting in a total of 149 studies.

### Characteristics of Included Studies

A detailed overview of study characteristics for PSD studies (*n* = 132) and PSA studies (*n* = 23) is presented in supplementary Online Resource 4 and 5. Of all PSD studies, 51 (39%) studies included only first-ever strokes. Thirty-nine cohorts (30%) were followed prospectively. Some studies used semi-structured psychiatric interviews like the Mini International Neuropsychiatric Interview (Sheehan et al. 1998), or the Structured Clinical Interview for DSM disorders (Spitzer et al. 1995), based on Diagnostic and Statistical Manual of Mental Disorders (DSM) version III (American Psychiatric Asociation 1980) or IV (American Psychiatric Asociation 1994), whereas others used clinician-rated or self-rated questionnaires (e.g. the Hamilton Depression Rating Scale (Hamilton 1960), Montgomery-Åsberg Depression Rating Scale (Montgomery and Asberg 1979), or the Geriatric Depression Scale (Yesavage et al. 1983) to evaluate the presence of PSD, and different cut-offs were applied.

Based on 107 (81%) studies that reported on PSD prevalence within the first year, a median prevalence of 30.4% was found (IQR 20.1–40.0). Of all PSA studies, nine (39%) studies included first-ever stroke patients. Four (17%) cohorts were studied prospectively. Most studies used the Apathy Scale (Starkstein et al. 1992) or Apathy Evaluation Scale (Marin et al. 1991) to evaluate the presence of PSA, and different cut-offs were applied. Based on 20 (87%) studies that reported on PSA prevalence within the first year, a median prevalence of 37.3% was found (IQR 22.1–42.5).

### Lesion Laterality

**Fig. 2:**
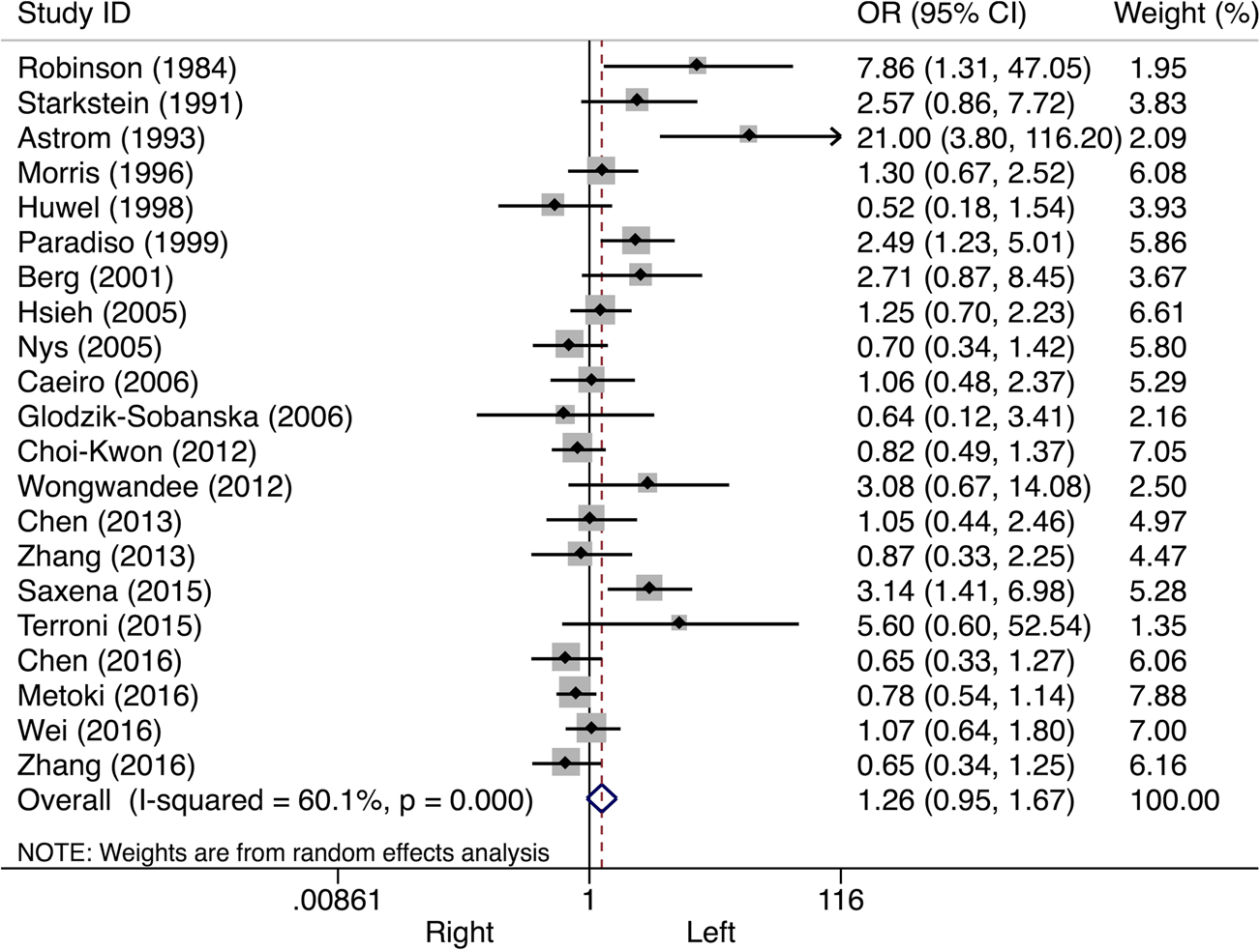
Forest plot of the relationship between post-stroke depression and lesion laterality. Subanalyses on acute stroke phase are presented. *CI* confidence interval, *OR* odds ratio

**Fig. 3:**
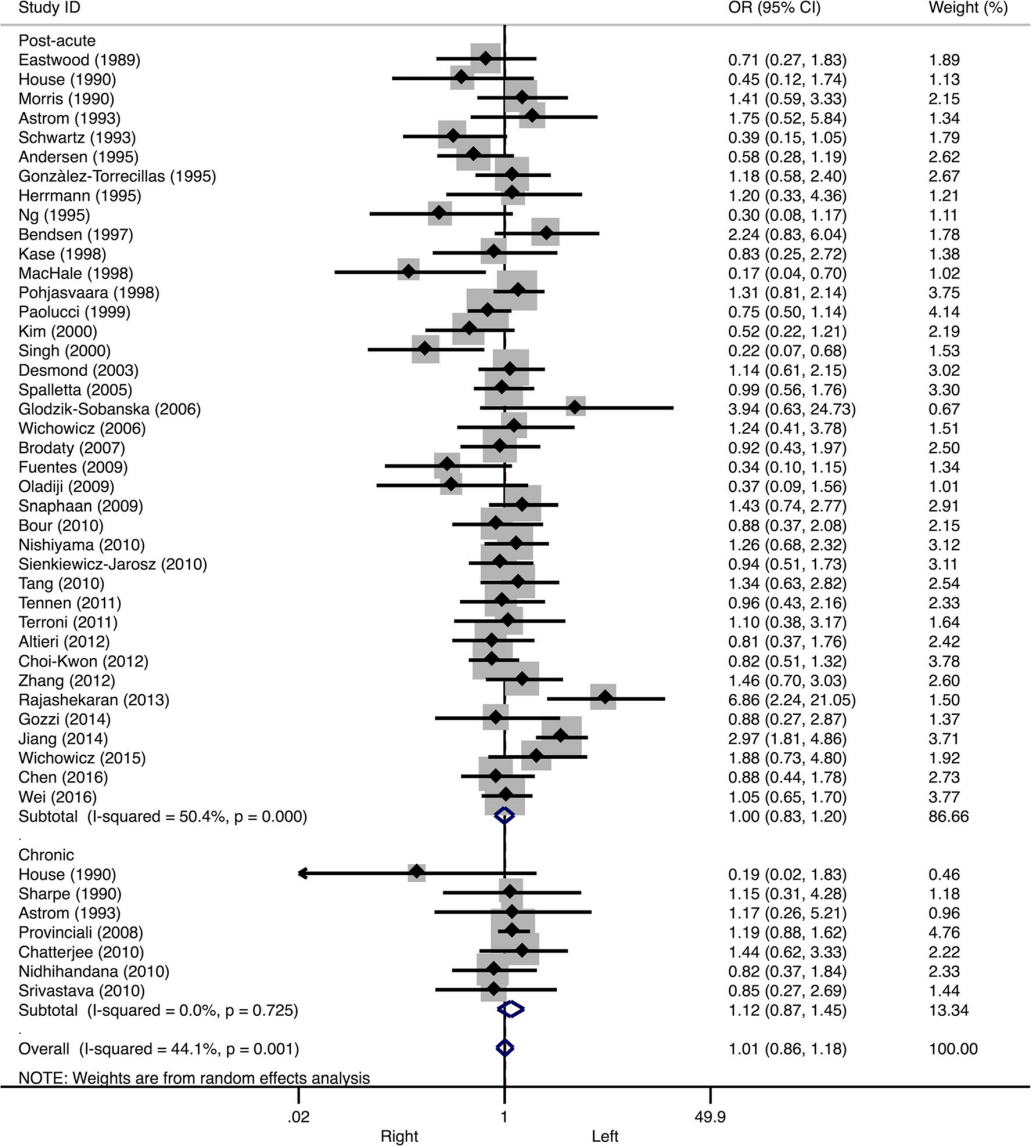
Forest plot of the relationship between post-stroke depression and lesion laterality. Subanalyses on post-acute stroke phase (*upper panels*) and chronic stroke phase (*lower panels*) are presented. *CI* confidence interval, *OR* odds ratio

Sixty (45%) studies presented data on PSD and lesion laterality. In the pooled analyses, no significant overall association between PSD and lesion side was found (Table 1). A subgroup analysis stratified by study phase showed a 26% higher odds of PSD after left-sided stroke in the acute phase, but this effect was not statistically significant (OR 1.26, 95% CI 0.95–1.67, *I* ^*2*^ = 60.1%, see Fig. 2). Neither in the post-acute stroke phase (OR 1.00, 95% CI 0.83–1.20, *I* ^*2*^ = 50.4%), nor in the chronic stroke phase (OR 1.12, CI 0.87–1.45, *I* ^*2*^ = 0.0%) a significant association was found with lesion side (see Fig. 3).

**Table 1:** Overall effect sizes and Egger’s bias coefficients

Marker	Number of studies included	Effect size		Heterogeneity		Publication bias	
		Odd’s ratio	95% CI	*I* ^*2*^ (%)	*p*-value	Egger’s bias coefficient	*p*-value
PSD							
Laterality	60^a^	1.07	0.93–1.23	49.6	< .001	0.14	.743
Type	14^b^	0.94	0.65–1.36	33.3	.102	−0.06	.943
Frontal lesions	30^c^	1.54	1.27–1.88	43.4	.004	0.92	.132
Subcortical lesions	10^d^	1.06	0.81–1.38	0.0	.601	1.00	.186
Basal ganglia lesions	12^e^	1.78	1.20–2.66	65.3	.001	0.24	.884
PSA							
Laterality	9	1.16	0.62–2.18	63.5	.005	1.69	.262
Type	4	0.82	0.19–3.53	75.2	.007	−3.92	.273
Frontal lesions	5	0.84	0.44–1.59	0.0	.635	2.71	.055
Subcortical lesions	2	1.03	0.38–2.80	0.0	.686	Not enough data	
Basal ganglia lesions	4	1.32	0.79–2.21	0.0	.891	−0.42	.536

*CI* confidence interval, *PSA* post-stroke apathy, *PSD* post-stroke depression

^a^Six of the 60 studies provided data on more than one time point

^b^One of the 14 studies provided data on more than one time point

^c^Five of the 30 studies provided data on more than one time point

^d^Two of the 10 studies provided data on more than one time point

^e^One of the 12 studies provided data on more than one time point

**Fig. 4:**
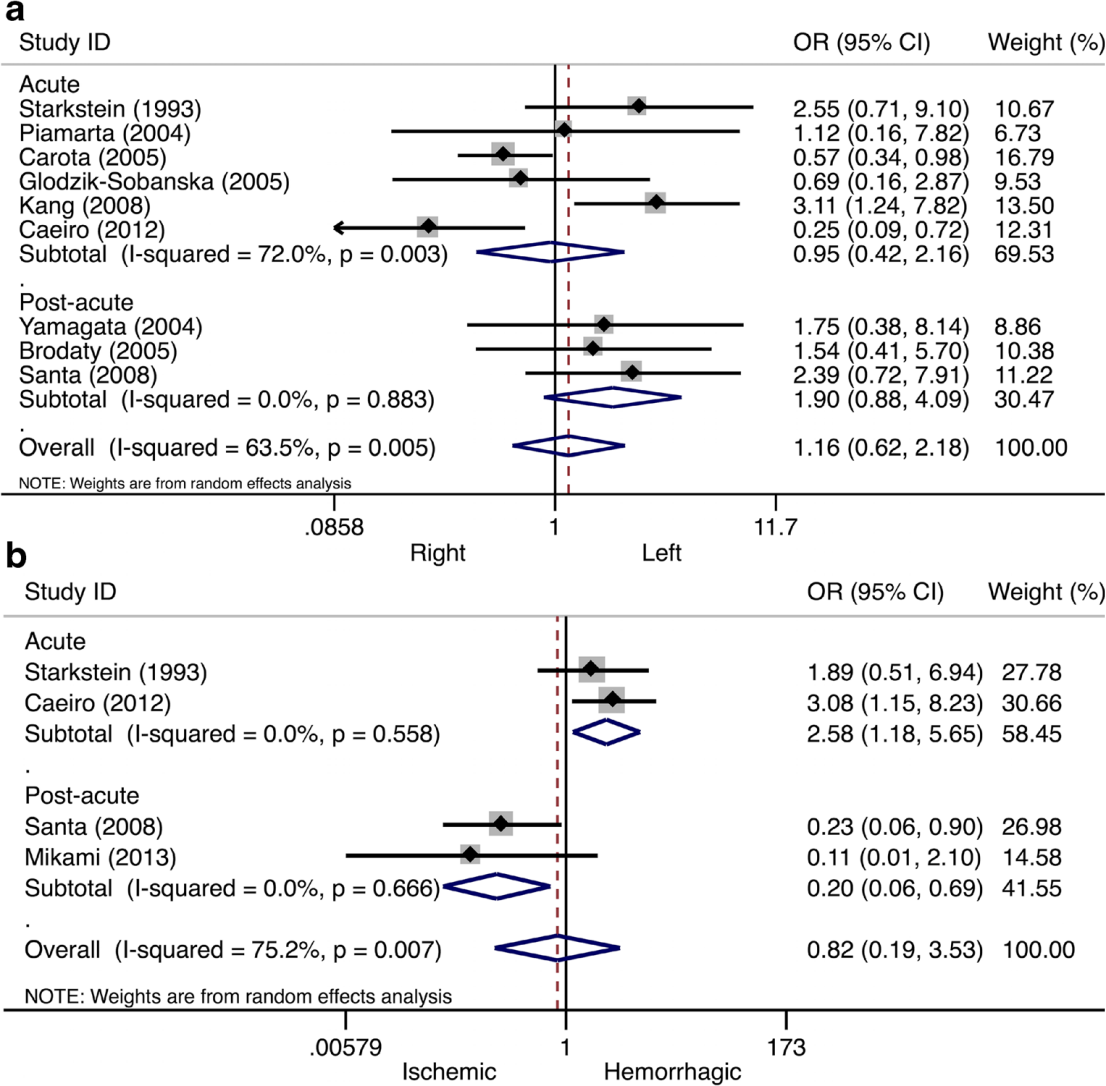
Forest plot of the relationship between post-stroke apathy and lesion laterality/type. In panel **a**, the results of the meta-analysis on lesion laterality are presented. In panel **b**, the results of the meta-analysis on lesion type are presented. Apart from the overall analysis, the subanalyses on acute stroke phase (*upper panels*) and post-acute stroke phase (*lower panels*) are presented. *CI* confidence interval, *OR* odds ratio

Nine (39%) studies presented data on PSA and lesion laterality. In the pooled analyses, the overall odds of PSA were a bit higher after left-sided stroke (Table 1). A subgroup analysis stratified by study phase showed higher odds after left-sided stroke in the post-acute phase, although this effect was not statistically significant (OR 1.90, 95% CI 0.88–4.09, *I* ^2^ = 0.0%, see Fig. 4a). No significant association was found in the acute stroke phase (OR 0.95, 95% CI 0.42–2.16, *I* ^2^ = 72.0%, see Fig. 4a) and no studies reported on the association in the chronic phase.

### Lesion Type

**Fig. 5:**
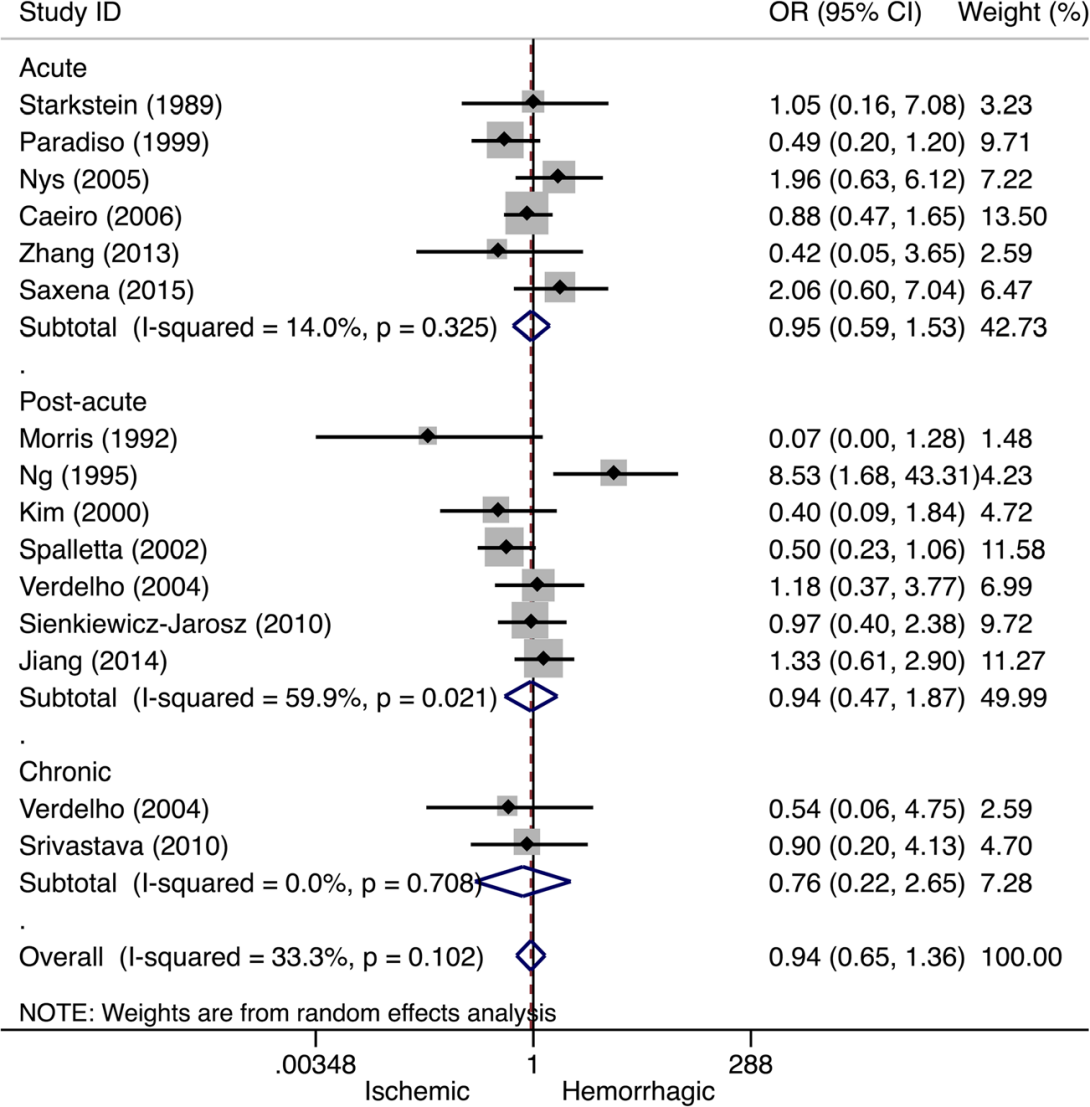
Forest plot of the relationship between post-stroke depression and lesion type. Apart from the overall analysis, the subanalyses on acute stroke phase (*upper panels*), post-acute stroke phase (*middle panels*), and chronic stroke phase (*lower panels*) are presented. *CI* confidence interval, *OR* odds ratio

Fourteen (11%) studies reported outcomes on lesion type associated with PSD. Overall, no significant association between PSD and lesion type was observed (Table 1). A subgroup analysis by study phase showed no significant association between lesion type and PSD in the acute (OR 0.95, 95% CI 0.59–1.53, *I* ^2^ = 14.0%), post-acute (OR 0.94, 95% CI 0.47–1.87, *I* ^2^ = 59.9%), or chronic stroke phase (OR 0.76, 95% CI 0.22–2.65, *I* ^2^ = 0.0%, see Fig. 5).

Four (17%) studies reported outcomes on lesion type associated with PSA. Overall, the odds of PSA after hemorrhagic stroke was not higher than after ischemic stroke (Table 1). A subgroup analysis by study phase showed higher odds after hemorrhagic stroke in the acute phase (OR 2.58, 95% CI 1.18–5.65, *I* ^*2*^ = 0.0%, see Fig. 4b), whereas higher odds after ischemic stroke were found in the post-acute phase (OR 0.20, 95% CI 0.06–0.69, *I* ^*2*^ = 0.0%, see Fig. 4b). Only two studies were included per phase.

### Lesion Location

**Fig. 6:**
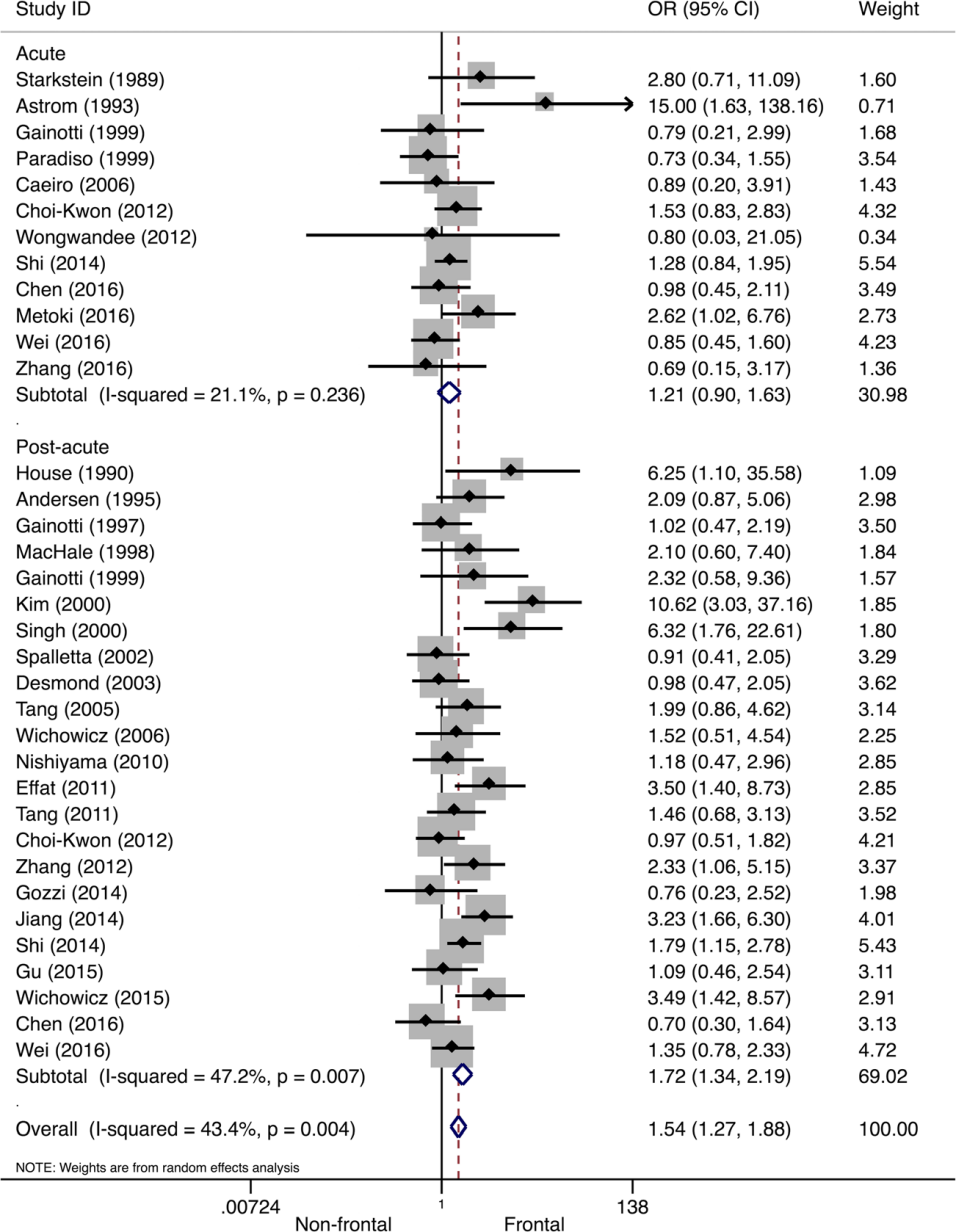
Forest plot of the relationship between post-stroke depression and frontal/anterior lesions. Apart from the overall analysis, the subanalyses on acute stroke phase (*upper panels*) and post-acute stroke phase (*lower panels*) are presented. *CI* confidence interval, *OR* odds ratio

In Table 2, an overview is provided of lesion locations that were significantly associated with PSD. As frontal/anterior, subcortical, and basal ganglia lesions were frequently associated with PSD, meta-analyses were performed on these locations. Thirty (23%) studies reported outcomes on frontal lesion location associated with PSD. Overall, a 54% higher odds of PSD after frontal stroke was found (Table 1). Subgroup analysis suggested this association was limited to PSD in the post-acute stroke phase (OR 1.72, 95% CI 1.34–2.19, *I* ^*2*^ = 47.2%), as no significant association was found in the acute stroke phase (OR 1.21, 95% CI 0.90–1.63, *I* ^*2*^ = 21.1%), see Fig. 6.

**Table 2:** Lesion locations significantly associated with post-stroke depression and post-stroke apathy

Lesion location		Studies	
	Acute phase	Post-acute phase	Chronic phase
PSD			
Anterior	Astrom et al. (1993); Herrmann et al. (1993); Shimoda and Robinson (1999)	Dam et al. (1989); House et al. (1990); Kim and Choi-Kwon (2000); Morris et al. (1992); Shimoda and Robinson (1999)	House et al. (1990)
Frontal lobe	Metoki et al. (2016); Robinson et al. (1984); Shi et al. (2014)	Aben et al. (2006); Effat et al. (2011); Hama et al. (2007b); Morris et al. (1996b); Murakami et al. (2013); Singh et al. (2000); Stojanovic and Stojanovic (2015); Tang et al. (2010); Wichowicz et al. (2015); Zhang et al. (2012)	–
Temporal lobe	Metoki et al. (2016); Terroni et al. (2015)	Zhang et al. (2012)	–
Posterior (occipital, parietal lobe)	Metoki et al. (2016); Paradiso and Robinson (1999); Starkstein et al. (1989)	Schwartz et al. (1993)	Shimoda and Robinson (1999)
Subcortical	Shi et al. (2014)	Schwartz et al. (1993); Tang et al. (2005); Zhang et al. (2012)	Chatterjee et al. (2010)
Basal ganglia	Herrmann et al. (1993); Yang et al. (2015b); Metoki et al. (2016)	Herrmann et al. (1995); Morris et al. (1996b); Nishiyama et al. (2010); Murakami et al. (2013); Wichowicz et al. (2015)	–
Insular cortex	Yang et al. (2015b)	–	–
Brainstem	–	Murakami et al. (2013)	–
Left hemisphere	Robinson et al. (1984); Robinson et al. (1985); Astrom et al. (1993); Morris et al. (1996a); Paradiso and Robinson (1999); Shimoda and Robinson (1999); Saxena and Suman (2015); Wongwandee et al. (2012)	Barker-Collo (2007); Jiang et al. (2014); Morris et al. (1996b); Rajashekaran et al. (2013); Wichowicz et al. (2015)	Parikh et al. (1988); Provinciali et al. (2008); Rashid et al. (2013); Stern and Bachman (1991)
Right hemisphere	Yang et al. (2015b)	Andersen et al. (1995); Castellanos-Pinedo et al. (2011); Dam et al. (1989); MacHale et al. (1998); Oladiji et al. (2009); Schwartz et al. (1993); Singh et al. (2000)	Stern and Bachman (1991); Verdelho et al. (2004)
Infratentorial	–	Iranmanesh and Vakilian (2009)	–
ACA	–	Desmond et al. (2003); Jiang et al. (2014); Tang et al. (2005)	Provinciali et al. (2008)
PCA	–	Desmond et al. (2003)	–
PSA			
Basal ganglia	Onoda et al. (2011)	Hama et al. (2007b); Mihalov et al. (2016); Murakami et al. (2013); Santa et al. (2008)	Rochat et al. (2013)
Thalamus	–	–	Rochat et al. (2013)
Pons / brainstem	–	Murakami et al. (2013); Tang et al. (2013a)	–
Right hemisphere	–	Castellanos-Pinedo et al. (2011)	–
Left hemisphere	Kang and Kim (2008)	–	–
Frontal lobe	Kang and Kim (2008)	–	–
CC / CG	Kang and Kim (2008)	–	–
IC (posterior limb)	Starkstein et al. (1993)	–	–

*ACA* anterior circulation area, *CC* corpus callosum, *CG* cingulate gyrus, *IC* internal capsule, *PCA* posterior circulation area, *PSA* post-stroke apathy, *PSD* post-stroke depression

**Fig. 7:**
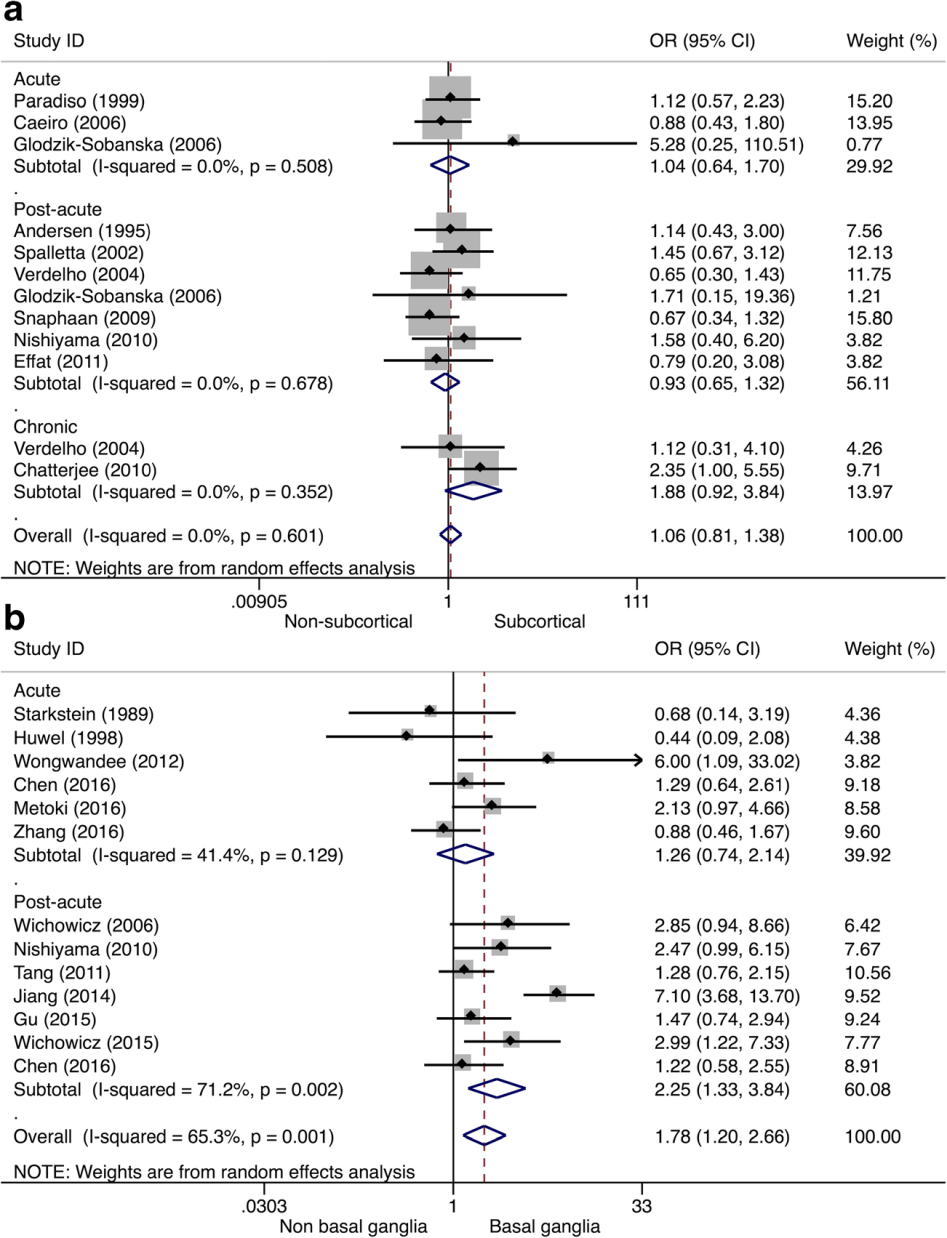
Forest plot of the relationship between post-stroke depression and subcortical/basal ganglia lesions. In panel **a**, the results of the meta-analysis on subcortical lesion location are presented. Apart from the overall analysis, the subanalyses on acute stroke phase (*upper panels*), post-acute stroke phase (*middle panels*), and chronic stroke phase (*lower panels*) are presented. In panel **b**, the results of the meta-analysis on basal ganglia lesions are presented. Apart from the overall analysis, the subanalyses on acute stroke phase (*upper panels*) and post-acute stroke phase (*lower panels*) are presented. *CI* confidence interval, *OR* odds ratio

Ten (8%) studies reported outcomes on subcortical lesion location associated with PSD. Pooled odds for PSD were not significantly higher after subcortical lesions (Table 1). A subgroup analysis by study phase showed no significant associations between subcortical lesions and PSD in the acute (OR 1.04, 95% CI 0.64–1.70), post-acute (OR 0.93, 95% CI 0.65–1.32), or chronic stroke phase (OR 1.88, 95% CI 0.92–3.84), see Fig. 7a), but the latter association consisted only of two studies. No significant heterogeneity was observed (each phase, *I* ^2^ = 0.0%). Twelve (9%) studies reported outcomes on basal ganglia lesion location associated with PSD. Overall, basal ganglia lesions were significantly associated with PSD (Table 1). A subgroup analysis by study phase showed that basal ganglia lesions were significantly associated with PSD in the post-acute phase (OR 2.25, 95% CI 1.33–3.84, *I* ^*2*^ = 71.2%), but not in the acute stroke phase (OR 1.26, 95% CI 0.74–2.14, *I* ^*2*^ = 41.4%), see Fig. 7b).

**Fig. 8:**
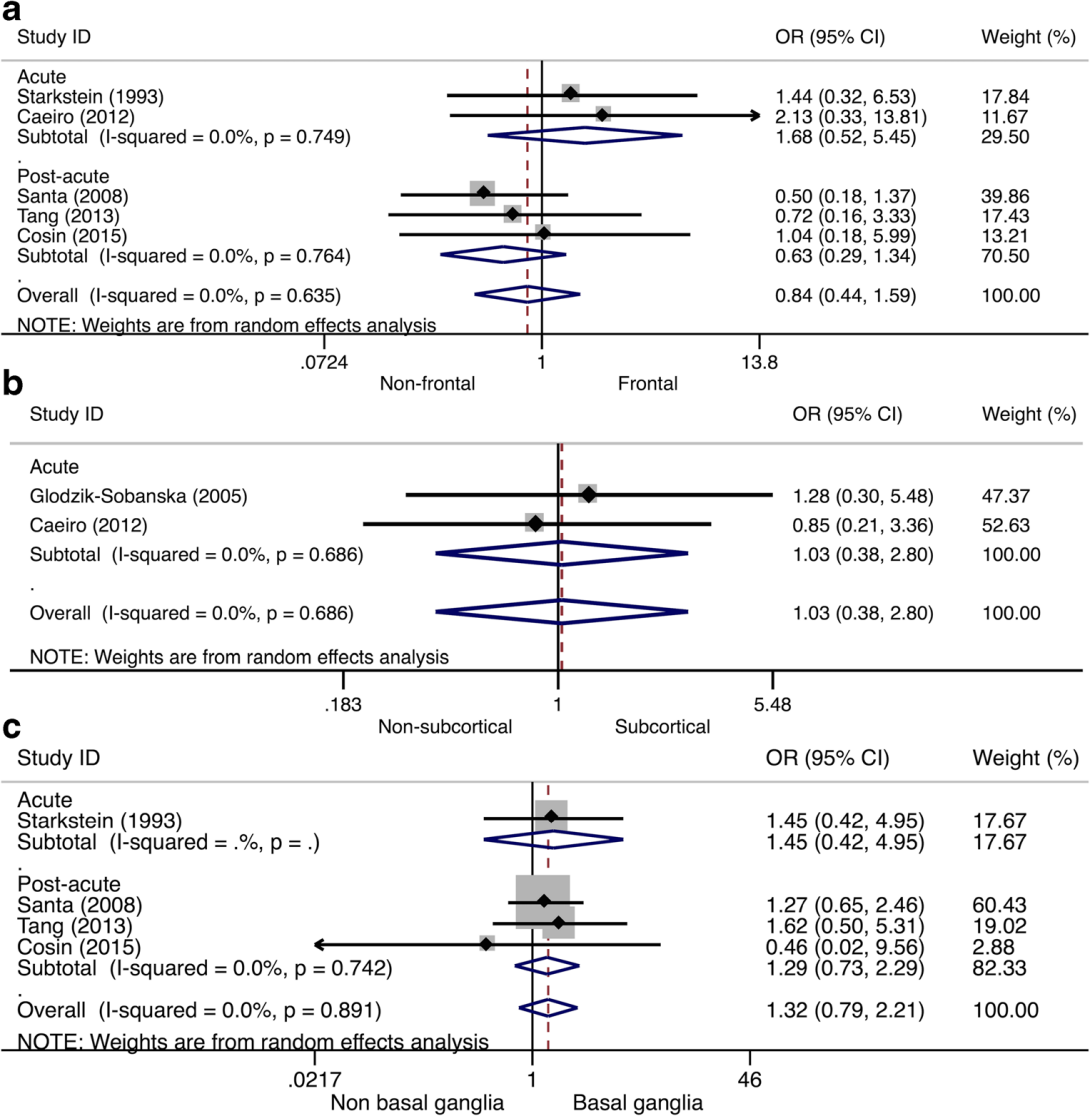
Forest plot of the relationship between post-stroke apathy and lesion location. In panel **a**, the results of the meta-analysis on frontal lesion location are presented. In panel **b**, the results of the meta-analysis on subcortical lesion location are presented. In panel **c**, the results of the meta-analysis on basal ganglia lesions are presented. Apart from the overall analysis, the subanalyses on acute stroke phase (*upper panels*) and post-acute stroke phase (*lower panels*) are presented. *CI* confidence interval, *OR* odds ratio

Five (22%) studies provided data on the association between PSA and frontal lesions. Overall, no significant association between PSA and frontal lesions was found (Table 1). Subgroup analyses showed different albeit no significant results per phase, with stronger associations with frontal lesions in the acute phase (OR 1.68, 95% CI 0.52–5.45, *I* ^*2*^ = 0.0%), and an inverse relation in the post-acute phase (OR 0.63, 95% CI 0.29–1.34, *I* ^*2*^ = 0.0%), see Fig. 8a. No significant association between PSA and subcortical lesions was found (Table 1), but this was only evaluated in two (9%) studies (OR 1.03, 95% CI 0.38–2.80, *I* ^*2*^ = 0.0%), see Fig. 8b. Four (17%) studies provided data on the association between PSA and basal ganglia lesions. Overall, no significant association between PSA and basal ganglia lesions was found (Table 1). Stratification by study phase showed similar results, with no significant heterogeneity (acute phase: OR 1.45, 95% CI 0.42–4.95, post-acute phase: OR 1.29, 95% CI 0.73–2.29), see Fig. 8c.

### Other Imaging Markers

Several studies examined imaging markers other than lesion location and type in association with PSD. These markers could not be evaluated in a meta-analysis. Therefore, the most important imaging markers are described qualitatively (see Table 3). PSD was associated with total (Chatterjee et al. 2010; Pavlovic et al. 2016), deep (Pavlovic et al. 2016), frontal (Chatterjee et al. 2010; Mok et al. 2010), and periventricular WMH (Pavlovic et al. 2016). Also, cerebral microbleeds are associated with PSD (Choi-Kwon et al. 2012; Tang et al. 2014a; Tang et al. 2011a, b, 2014b), and several studies showed that PSD is more prevalent in patients with a large lesion volume (Hama et al. 2007b; Ku et al. 2013; MacHale et al. 1998; Morris et al. 1992; Nys et al. 2005; Schwartz et al. 1993; Sharpe et al. 1990, 1994; Shimoda and Robinson 1999; Zhang et al. 2012) or large number of lesions (Bendsen et al. 1997; Chatterjee et al. 2010; Jiang et al. 2014; Pavlovic et al. 2016; Tang et al. 2014b; Zhang et al. 2012).

**Table 3:** Imaging markers associated with post-stroke depression and post-stroke apathy

Imaging marker		Studies	
	Acute phase	Post-acute phase	Chronic phase
PSD			
Degree of WMH	–	Deep WMH: Kim et al. (2011); Tang et al. (2010)	Overall, BG, frontal WMC: Chatterjee et al. (2010)
		Left frontal WMH: Mok et al. (2010)	Overall, deep, periventricular WMH: Pavlovic et al. (2016)
Cerebral microbleeds	Choi-Kwon et al. (2012)	Tang et al. (2011a, b, 2014a, b)	–
Large lesion volume	Shimoda and Robinson (1999); Ku et al. (2013); Nys et al. (2005)	Hama et al. (2007b); MacHale et al. (1998); Morris et al. (1992); Schwartz et al. (1993); Shimoda and Robinson (1999); Zhang et al. (2012)	Sharpe et al. (1990, 1994); Shimoda and Robinson (1999)
Large number of lesions	–	Bendsen et al. (1997); Jiang et al. (2014); Tang et al. (2014b); Zhang et al. (2012)	Chatterjee et al. (2010); Lacunar lesions: Pavlovic et al. (2016)
Metabolism	Huang et al. (2010); Xu et al. (2008)	Glodzik-Sobanska et al. (2006); Wang et al. (2012); Xu et al. (2008)	–
Atrophy	–	Left IFG: Fu et al. (2010) FL: Tang et al. (2013b)	Subcortical: Astrom et al. (1993); Starkstein et al. (1988)
Regional cerebral blood flow	–	Left hemisphere: Wichowicz et al. (2006)	–
Functional connectivity / fractional anisotropy	Altered FC in left orbital part of IFG: Zhang et al. (2014)	Frontal WM integrity: Williamson et al. (2010); Increased ratio FA values in bilateral anterior limbs of IC: Yasuno et al. (2014)	–
PSA			
Degree of WMH	–	RH WMH, right fronto-subcortical circuit WMH: Brodaty et al. (2005); Periventricular WMH: Tang et al. (2013a)	–
Large lesion volume	–	Hama et al. (2007b)	–
Large number of lesions	–	Tang et al. (2013a)	–
Metabolism	Glodzik-Sobanska et al. (2005)	–	–
Regional cerebral blood flow	Bilateral BG: Onoda et al. (2011)	–	Right dlF and lFT: Okada et al. (1997)
Fractional anisotropy	–	Reduced FA in Genu of CC, left anterior corona radiata, splenium of CC, and WM in the right IFG: Yang et al. (2015c). Reduced median FA, reduction in WM integrity in anterior cingulum, fornix and uncinate fasciculus: Hollocks et al. (2015)	–
Atrophy	–	Frontal cortical atrophy: Mihalov et al. (2016)	–

*ATR* atrophy, *BG* basal ganglia, *CC* corpus collosum, *CMB* cerebral microbleeds, *dlF* dorsolateral frontal, *FA* fractional anisotropy, *FC* functional connectivity, *FL* frontal lobe, *IFG* inferior frontal gyrus, *lFT* left frontotemporal, *RH* right hemisphere, *WM* white matter, *WMC* white matter changes, *WMH* white matter hyperintensities, *PSA* post-stroke apathy, *PSD* post-stroke depression

More recently, advanced diffusion tensor imaging (DTI) techniques have been used to investigate the association between microstructural abnormalities in white matter (WM) and PSD. Yasuno et al. (2014) showed that a reduction in fractional anisotropy (FA) in the bilateral anterior limbs of the internal capsule was associated with an increased risk of PSD and Williamson et al. (2010) showed that decreased WM integrity in the frontal lobes was associated with mood deficits. This indicates that WM damage in certain brain regions is associated with the development of PSD. A resting-state functional MRI (fMRI) study showed that altered functional connectivity in regions involved in affect was associated with higher levels of depression (Zhang et al. 2014). Atrophy also seems to be an important predictor of PSD, as significant associations were found with frontal lobe atrophy (Tang et al. 2013b), subcortical atrophy (Astrom et al. 1993; Starkstein et al. 1988), and left inferior frontal gyrus atrophy (Fu et al. 2010). Interestingly, none of these studies reported on hippocampal atrophy. Recently, Chen et al. (2016) looked at medial temporal lobe atrophy, but found no association with PSD in the acute or post-acute stroke phase. According to proton magnetic resonance spectroscopy (^1^H–MRS) studies, biochemical changes in metabolite levels in frontal lobe (Glodzik-Sobanska et al. 2006; Wang et al. 2012; Xu et al. 2008), hippocampus (Huang et al. 2010), and left thalamus (Huang et al. 2010) seem to accompany the development of PSD.

Compared with PSD studies, only few studies evaluated imaging markers related to PSA (see Table 3). PSA was significantly associated with degree of right-hemisphere (Brodaty et al. 2005), right fronto-subcortical circuit (Brodaty et al. 2005), and periventricular WMH (Tang et al. 2013a). In addition, large lesion volume (Hama et al. 2007b), and large number of lesions (Tang et al. 2013a) were associated with PSA. A recent study by Mihalov et al. (2016) showed that frontal cortical atrophy was a strong predictor of PSA, and this relation increased with higher age. In two DTI studies reductions in FA in several brain areas were associated with an increased level of apathy (Yang et al. 2015c). In addition, PSA was associated with reductions in regional cerebral blood flow in the bilateral basal ganglia (Onoda et al. 2011), right dorsolateral frontal cortex, and left frontotemporal cortex (Okada et al. 1997) measured with single-photon emission computed tomography. An H^1^-MRS study suggested that lower N-acetylaspartate/creatine ratio in the right frontal lobe was related to PSA (Glodzik-Sobanska et al. 2005).

### Meta-Regression Analyses

Egger’s regression tests showed no evidence for statistically significant small-study effects in above meta-analyses (see Table 1), although it was not possible to calculate Egger’s regression coefficients for the association with subcortical lesions in PSA as the pooled sample size was too small. Visual inspection of the shape of the funnel plots also did not reveal convincing evidence of obvious asymmetry (see supplementary Online Resource 6). However, some plots, especially for the PSA studies, only consisted of few studies.

Meta-regression analyses were performed to assess potential sources of heterogeneity between PSD studies reporting on lesion laterality (*n* = 60). None of the included variables appeared to be a significant cause of heterogeneity. In addition, meta-regression analyses were performed on PSD studies reporting on frontal (*n* = 30) and basal ganglia lesions (*n* = 12). Only study phase appeared to be a significant cause of heterogeneity in both analyses (frontal: *p* = 0.041, residual *I* ^*2*^ = 58.9%, Adj. *R* ^*2*^ = 25.7%; basal ganglia: *p* = 0.044, residual *I* ^*2*^ = 55.4%, Adj. *R* ^*2*^ = 50.7%). To assess potential sources of heterogeneity among PSA studies reporting on lesion laterality (*n* = 9), meta-regression analyses were performed showing that only imaging method appeared to be an important cause of heterogeneity (*p* = 0.052, residual *I* ^*2*^ = 31.2%, Adj. *R* ^*2*^ = 64.6%).

## Discussion

This systematic review and meta-analysis summarizes the most up-to-date information on a range of imaging markers associated with PSD and PSA during the acute, post-acute, and chronic stroke phase. Meta-analyses indicated that PSD in the post-acute phase was significantly more frequent in patients with frontal or basal ganglia lesions. No significant association was found between PSD and lesion laterality in the post-acute and chronic stroke phase. Nevertheless, it is of interest to mention that left-sided stroke occurred more often in the PSD group in the acute phase. This result became insignificant after the inclusion of four recent large studies (Chen et al. 2016; Metoki et al. 2016; Wei et al. 2016; Zhang et al. 2016), which differed from the other studies in that they reported a relatively low PSD prevalence (median 18.6%, IQR 17.4–30.2). Frequency of PSD was equal for ischemic and hemorrhagic stroke in all stroke phases, but PSA was more frequent after hemorrhagic stroke in the acute phase, whereas it was more frequent after ischemic stroke in the post-acute phase. Since only four PSA studies were available, this finding should be interpreted with caution. Also, PSA did not depend on lesion laterality or location, but again the amount of available PSA studies was small in general.

Our meta-analysis updates and extends previous studies. The meta-analysis by Wei et al. (2015) on lesion laterality and PSD found a significant association between right hemispheric lesions and risk of PSD in the post-acute stroke phase (1–6 months). In contrast to Wei et al. (2015), we defined the post-acute period as 15 days to 6 months, which could explain the difference in results. In agreement with Caeiro et al. (2013a) the prevalence of PSA was not associated with lesion laterality. Both meta-analyses did not study associations with markers other than lesion laterality and lesion type, while the review of van Dalen et al. (2013) evaluated associations between PSA and lesion location only qualitatively and concluded that no clear association could be found.

The present findings suggest that lesion location is an important risk factor for PSD in the post-acute stroke phase. However, in the past few years the hypothesis of PSD and PSA being associated with damage to specific lesion locations has been shifted to the idea that damage to a neuronal network involved in affect is underlying the development of PSD and PSA (Tang et al. 2011c; Terroni et al. 2011; Vataja et al. 2001), with different sub-circuits involved in PSD (Yang et al. 2015b) and PSA (Yang et al. 2015a), see Table 4. DTI is a promising tool to identify more accurately how these brain networks are affected after stroke. The qualitative overview of imaging markers associated with PSD and PSA showed that not only direct stroke-related features such as lesion location, lesion volume, and number of lesions, but also other neurovascular, non-directly stroke-related but often co-occurring features, such as degree of WMH, cerebral microbleeds, and atrophy, were frequently associated with PSD. With respect to PSA, associations with degree of WMH, lesion volume, and number of lesions were found in some extent. Co-occurring vascular lesions may make a stroke patient more vulnerable for developing PSD and PSA. Therefore, future studies should focus on a broader range of imaging markers, including lesion volume, atrophy, WMH, and cerebral microbleeds, and also how lesion-related markers may interact with co-occuring indirect vascular markers. Besides, advanced imaging techniques (e.g. DTI, fMRI) are needed to evaluate how microstructural abnormalities and changes in functional connectivity contribute to the development of PSD and PSA.

**Table 4:** Circuits associated with post-stroke depression and post-stroke apathy

Studies	Phase	Circuits - network
PSD		
Terroni et al. (2011)	Acute	Disruption of limbic-cortical-striatal-pallidal-thalamic circuit, Medial PFC dysfunction
Yang et al. (2015b)	Acute	Frontal lobe, insula, limbic system, parietal lobe, basal ganglia, temporal lobe
Vataja et al. (2001)	Post-acute	Higher number and lesion volume in (left) prefronto-subcortical circuit
Tang et al. (2011c)	Post-acute	Lesions in frontal subcortical circuits
PSA		
Yang et al. (2015a)	Acute	Limbic system, basal ganglia, insula, frontal, temporal, parietal, occipital lobe

*PFC* prefrontal cortex, *PSA* post-stroke apathy, *PSD* post-stroke depression

Our study has the following strengths. A large amount of publications on PSD were identified, resulting in a rich pooled cohort of studies that were not included in earlier meta-analyses (Chen et al. 2013, 2016; Gozzi et al. 2014; Jiang et al. 2014; Metoki et al. 2016; Saxena and Suman 2015; Terroni et al. 2015; Wei et al. 2016; Wichowicz et al. 2015; Zhang et al. 2016). Furthermore, beside information on lesion laterality, also data on other imaging markers was retrieved for quantitative and qualitative analysis. Therefore, the present review provides an up-to-date and extended overview of findings on the association between imaging markers and risk of PSD and PSA.

One limitation of the present study was the small amount of studies on PSA, which made it difficult to perform sub-analyses. Therefore, future studies are needed on imaging markers of PSA, covering a broad range of imaging markers. Nevertheless, as heterogeneity was small between PSA studies, we believe that the results are still of importance, but should be interpreted with caution as the generalizability and validity is compromised in comparison with meta-analyses including a larger amount of studies. In addition, moderate to high (unexplained) heterogeneity between studies in some meta-analyses indicated large differences in methodology between studies. Particularly the use of different scales and cut-offs to define the presence of depression and apathy and different imaging methods (CT vs. MRI) are of influence on the comparability of findings. Also differences in eligibility criteria (e.g. exclusion of patients with aphasia, differences in age range) can create heterogeneity among studies. Meta-regression analyses were performed to identify potential sources of heterogeneity, and only study phase for PSD studies and imaging method for PSA studies could be identified. However, in addition to the included variables, also other potential variables (e.g. years of education, cognitive status), that could not be included in the analyses due to the large variability in the methods and availability of data between studies, might explain some of the between-study difference in effect estimates. Therefore, we performed random-effects meta-analyses, which take the heterogeneity between studies into account.

### Conclusion

The present study suggests that lesion location rather than lesion laterality or type may be an important risk factor for PSD in the post-acute stroke phase. In contrast, lesion type rather than lesion laterality or location might be an important factor in determining who is at risk to develop PSA in the acute and post-acute phase, though additional studies are needed to confirm this, as the sample size was small. Therefore, large multicenter cohort studies using advanced imaging techniques and focusing on both PSD and PSA from the acute to the chronic stroke phase are strongly needed.

## Electronic supplementary material

ESM 1 (DOCX 125 kb)

ESM 2 (DOCX 143 kb)

ESM 3 (DOCX 147 kb)

ESM 4 (DOCX 169 kb)

ESM 5 (DOCX 135 kb)

ESM 6 (DOCX 347 kb)

ESM 7 (DOC 63 kb)
