# Supplementary material for: Imaging Markers of Post-Stroke Depression and Apathy: a Systematic Review and Meta-Analysis
Source: Neuropsychol Rev. 2017 Aug 22;27(3):202–19. doi: 10.1007/s11065-017-9356-2 (PMC5613051; doi:10.1007/s11065-017-9356-2)
Supplement: Supplementary file 1 — (DOCX 125 kb) [file 11065_2017_9356_MOESM1_ESM.docx]

Supplementary Table 1

| Medline | Platform | OvidSP |
| --- | --- | --- |
|  | Database | MEDLINE® In-Process & Other Non-Indexed Citations |
|  | Limits | Human subjects, adult subjects, language (German, Dutch, English, French) |
|  | Search | (ischem* OR ischaem* OR stroke OR infarct* OR hemorrhag* OR haemorrhag* OR  lesion).ti,kf,hw  AND  (apath* OR depress* OR mood OR motivation OR neuropsychia* OR emotion OR abulia).ti,kf,hw  AND  (MRI OR (magnetic AND resonance) OR imaging OR SPECT OR (Single-photon AND Emission AND Positron AND Computed AND Tomography) OR fMRI OR (functional AND MRI) OR PET OR (Positron AND Emission AND Tomography) OR CT OR (Computed AND tomography) OR DTI OR (diffusion AND tensor) OR connectivity).mp |
| Embase | Platform | OvidSP |
|  | Database | Embase 1980-present |
|  | Limits | Human subjects, adult subjects, language (German, Dutch, English, French) |
|  | Search | (ischem* OR ischaem* OR stroke OR infarct* OR hemorrhag* OR haemorrhag* OR lesion).ti,kw,hw  AND  (apath* OR depress* OR mood OR motivation OR neuropsychia* OR emotion OR abulia).ti,kw,hw  AND  (MRI OR (magnetic AND resonance) OR imaging OR SPECT OR (Single-photon AND Emission AND Positron AND Computed AND Tomography) OR fMRI OR (functional AND MRI) OR PET OR (Positron AND Emission AND Tomography) OR CT OR (Computed AND tomography) OR DTI OR (diffusion AND tensor) OR connectivity).mp |
| PsycINFO | Platform | EBSCOhost |
|  | Database | PsycINFO 1806 - present |
|  | Limits | Human subjects, adult subjects, language (German, Dutch, English, French) |
|  | Search | (ischem* OR ischaem* OR stroke OR infarct* OR haemorrhag* OR hemorrhage* or lesion)SU  AND  (apath* OR depress* OR mood OR motivation OR neuropsychia* OR emotion OR abulia)SU  AND  (MRI OR magnetic OR resonance OR imaging OR computed OR CT)TX |
| CINAHL | Platform | EBSCOhost |
|  | Database | CINAHL 1981 - present |
|  | Limits | Human subjects, adult subjects, language (German, Dutch, English, French) |
|  | Search | (ischem* OR ischaem* OR stroke OR infarct* OR hemorrhage* OR haemorrhag* OR lesion)SU  AND  (apath* OR depress* OR mood OR motivation OR neuropsychia* OR emotion OR abulia)SU  AND  (MRI OR magnetic OR resonance OR imaging OR computed OR CT)TX |
| Cochrane | Platform | Cochrane |
|  | Database | Wiley Online Library |
|  | Limits | Human subjects, adult subjects, language (German, Dutch, English, French) |
|  | Search | (ischem* OR ischaem* OR stroke OR infarct* OR haemorrhag* OR hemorrhage* OR lesion) AND (apath* OR depress* OR mood OR motivation OR neuropsychia* OR emotion OR abulia) AND (MRI OR (magnetic AND resonance) OR imaging OR CT OR (Computed AND tomography) OR DTI OR (diffusion AND tensor) OR connectivity) TITLE/ABSTRACT/KEYWORDS |

Imaging markers of post-stroke depression and apathy: a systematic review and meta-analysis

Elles Douven,^1^ Sebastian Köhler,^1^ Maria M.F. Rodriguez,^2^ Julie Staals,^3^ Frans R.J. Verhey,^1^ and Pauline Aalten^1*^

^1.^ Alzheimer Center Limburg, School for Mental Health and Neuroscience (MHeNS), Maastricht University Medical Center (MUMC+), Maastricht, The Netherlands.

^2.^ Complexo Universitario de Vigo, Hospital Alvaro Cunqueiro. Department of Psychiatry, Vigo, Spain.

^3.^ Department of Neurology, Cardiovascular Research Institute Maastricht (CARIM), MUMC+, Maastricht, The Netherlands.
