# Supplementary material for: Imaging Markers of Post-Stroke Depression and Apathy: a Systematic Review and Meta-Analysis
Source: Neuropsychol Rev. 2017 Aug 22;27(3):202–19. doi: 10.1007/s11065-017-9356-2 (PMC5613051; doi:10.1007/s11065-017-9356-2)
Supplement: Supplementary file 2 — (DOCX 143 kb) [file 11065_2017_9356_MOESM2_ESM.docx]

Supplementary Table 2 Contingency table data of meta-analyses separate for post-stroke depression and post-stroke apathy studies

| *PSD studies* | | | | | | | | | | | | | | | | | | | | |
| --- | --- | --- | --- | --- | --- | --- | --- | --- | --- | --- | --- | --- | --- | --- | --- | --- | --- | --- | --- | --- |
|  | Laterality | | | | Lesion type | | | | Frontal lesion | | | | Subcortical lesion | | | | Basal ganglia lesion | | | |
| Authors | PSD | | No-PSD | | PSD | | No-PSD | | PSD | | No-PSD | | PSD | | No-PSD | | PSD | | No-PSD | |
|  | Left | Right | Left | Right | Hem | Isch | Hem | Isch | Fr | NFr | Fr | NFr | Subc | NSubc | Subc | Nsubc | BG | nBG | BG | nBG |
| Acute stroke phase | | | | | | | | | | | | | | | | | | | | |
| Robinson et al. (1984a) | 11 | 2 | 7 | 10 | - | - | - | - | - | - | - | - | - | - | - | - | - | - | - | - |
| Starkstein et al. (1989) | - | - | - | - | 2 | 14 | 3 | 22 | 7 | 10 | 5 | 20 | - | - | - | - | 3 | 14 | 6 | 19 |
| Starkstein et al. (1991) | 18 | 8 | 14 | 16 | - | - | - | - | - | - | - | - | - | - | - | - | - | - | - | - |
| Astrom et al. (1993)^*^ | 14 | 2 | 7 | 21 | - | - | - | - | 12 | 2 | 2 | 5 | - | - | - | - | - | - | - | - |
| Morris et al. (1996a) | 21 | 26 | 56 | 90 | - | - | - | - | - | - | - | - | - | - | - | - | - | - | - | - |
| Huwel et al. (1998) | 12 | 18 | 14 | 11 | - | - | - | - | - | - | - | - | - | - | - | - | 3 | 27 | 5 | 20 |
| Gainotti et al. (1999) | - | - | - | - | - | - | - | - | 4 | 11 | 12 | 26 | - | - | - | - | - | - | - | - |
| Paradiso et al. (1999) | 32 | 22 | 31 | 53 | 8 | 46 | 22 | 62 | 37 | 17 | 63 | 21 | 26 | 28 | 38 | 46 | - | - | - | - |
| Berg et al. (2001) | 11 | 6 | 21 | 31 | - | - | - | - | - | - | - | - | - | - | - | - | - | - | - | - |
| Hsieh and Kao (2005) | 32 | 39 | 54 | 82 | - | - | - | - | - | - | - | - | - | - | - | - | - | - | - | - |
| Nys et al. (2005) | 25 | 41 | 28 | 32 | 10 | 56 | 5 | 55 | - | - | - | - | - | - | - | - | - | - | - | - |
| Glodzik-Sobanska et al. (2006)^*^ | 4 | 4 | 11 | 7 | - | - | - | - | - | - | - | - | 8 | 0 | 14 | 4 | - | - | - | - |
| Caeiro et al. (2006) | 21 | 26 | 22 | 29 | 25 | 57 | 32 | 64 | 5 | 7 | 8 | 10 | 17 | 65 | 22 | 74 | - | - | - | - |
| Wongwandee (2012) | 8 | 3 | 13 | 15 | - | - | - | - | 0 | 11 | 1 | 27 | - | - | - | - | 9 | 2 | 12 | 16 |
| Choi-Kwon et al. (2012)^*^ | 29 | 41 | 203 | 235 | - | - | - | - | 16 | 54 | 71 | 367 | - | - | - | - | - | - | - | - |
| Chen et al. (2013) | 13 | 18 | 29 | 42 | - | - | - | - | - | - | - | - | - | - | - | - | - | - | - | - |
| Zhang et al. (2013) | 9 | 16 | 26 | 40 | 1 | 24 | 6 | 60 | - | - | - | - | - | - | - | - | - | - | - | - |
| Shi et al. (2014) | - | - | - | - | - | - | - | - | 36 | 267 | 73 | 691 | - | - | - | - | - | - | - | - |
| Terroni et al. (2015) | 6 | 1 | 15 | 14 | - | - | - | - | - | - | - | - | - | - | - | - | - | - | - | - |
| Saxena and Suman (2015) | 42 | 19 | 19 | 27 | 10 | 51 | 4 | 42 | - | - | - | - | - | - | - | - | - | - | - | - |
| Metoki et al. (2016) | 59 | 85 | 220 | 248 | - | - | - | - | 7 | 64 | 14 | 336 | - | - | - | - | 10 | 61 | 25 | 325 |
| Chen et al. (2016)^*^ | 29 | 33 | 46 | 34 | - | - | - | - | 13 | 72 | 19 | 103 | - | - | - | - | 18 | 67 | 21 | 101 |
| Wei et al. (2016)^*^ | 33 | 38 | 133 | 164 | - | - | - | - | 15 | 56 | 71 | 226 | - | - | - | - | - | - | - | - |
| Zhang et al. (2016) | 19 | 26 | 109 | 97 | - | - | - | - | 2 | 43 | 13 | 193 | - | - | - | - | 21 | 24 | 103 | 103 |
| Post-acute stroke phase | | | | | | | | | | | | | | | | | | | | |
| Eastwood et al. (1989) | 14 | 33 | 12 | 20 | - | - | - | - | - | - | - | - | - | - | - | - | - | - | - | - |
| Morris et al. (1990) | 18 | 16 | 24 | 30 | 0 | 14 | 7 | 14 | - | - | - | - | - | - | - | - | - | - | - | - |
| House et al. (1990)^*^ | 4 | 7 | 29 | 23 | - | - | - | - | 5 | 2 | 16 | 40 | - | - | - | - | - | - | - | - |
| Morris et al. (1992) | - | - | - | - | - | - | - | - | - | - | - | - | - | - | - | - | - | - | - | - |
| Schwartz et al. (1993) | 7 | 29 | 21 | 34 | - | - | - | - | - | - | - | - | - | - | - | - | - | - | - | - |
| Astrom et al. (1993)^*^ | 11 | 8 | 11 | 14 | - | - | - | - | - | - | - | - | - | - | - | - | - | - | - | - |
| González-Torrecillas et al. (1995) | 23 | 25 | 36 | 46 | - | - | - | - | - | - | - | - | - | - | - | - | - | - | - | - |
| Herrmann et al. (1995) | 12 | 5 | 20 | 10 | - | - | - | - | - | - | - | - | - | - | - | - | - | - | - | - |
| Andersen et al. (1995) | 15 | 22 | 84 | 71 | - | - | - | - | 12 | 16 | 24 | 67 | 21 | 7 | 66 | 25 | - | - | - | - |
| Ng et al. (1995) | 4 | 25 | 8 | 15 | 13 | 16 | 2 | 21 | - | - | - | - | - | - | - | - | - | - | - | - |
| Bendsen et al. (1997) | 13 | 7 | 49 | 59 | - | - | - | - | - | - | - | - | - | - | - | - | - | - | - | - |
| Gainotti et al. (1997) | - | - | - | - | - | - | - | - | 16 | 34 | 24 | 52 | - | - | - | - | - | - | - | - |
| MacHale et al. (1998) | 3 | 10 | 27 | 15 | - | - | - | - | 7 | 6 | 15 | 27 | - | - | - | - | - | - | - | - |
| Kase et al. (1998) | 10 | 11 | 12 | 11 | - | - | - | - | - | - | - | - | - | - | - | - | - | - | - | - |
| Pohjasvaara et al. (1998) | 65 | 46 | 86 | 80 | - | - | - | - | - | - | - | - | - | - | - | - | - | - | - | - |
| Paolucci et al. (1999) | 51 | 75 | 161 | 178 | - | - | - | - | 41 | 85 | 124 | 215 | - | - | - | - | - | - | - | - |
| Gainotti et al. (1999) | - | - | - | - | - | - | - | - | 5 | 7 | 8 | 26 | - | - | - | - | - | - | - | - |
| Singh et al. (2000) | 5 | 24 | 25 | 27 | - | - | - | - | 10 | 19 | 4 | 48 | - | - | - | - | - | - | - | - |
| Kim et al. (2000) | 11 | 16 | 69 | 52 | 2 | 25 | 20 | 101 | 24 | 3 | 52 | 69 | - | - | - | - | - | - | - | - |
| Gainotti et al. (2001) | - | - | - | - | - | - | - | - | - | - | - | - | - | - | - | - | - | - | - | - |
| Spalletta et al. (2002) | - | - | - | - | 16 | 72 | 20 | 45 | 21 | 51 | 14 | 31 | 32 | 40 | 16 | 29 | - | - | - | - |
| Desmond et al. (2003) | 17 | 30 | 124 | 250 | - | - | - | - | 10 | 37 | 81 | 293 | - | - | - | - | - | - | - | - |
| Verdelho et al. (2004)^*^ | - | - | - | - | 6 | 40 | 7 | 55 | - | - | - | - | 25 | 21 | 40 | 22 | - | - | - | - |
| Spalletta et al. (2005) | 43 | 69 | 34 | 54 | - | - | - | - | - | - | - | - | - | - | - | - | - | - | - | - |
| Tang et al. (2005) | - | - | - | - | - | - | - | - | 17 | 10 | 71 | 83 | - | - | - | - | - | - | - | - |
| Wichowicz et al. (2006) | 16 | 7 | 24 | 13 | - | - | - | - | 9 | 14 | 11 | 26 | - | - | - | - | 11 | 12 | 9 | 28 |
| Glodzik-Sobanska et al. (2006)^*^ | 7 | 2 | 8 | 9 | - | - | - | - | - | - | - | - | 8 | 1 | 14 | 3 | - | - | - | - |
| Brodaty et al. (2007) | 17 | 20 | 47 | 51 | - | - | - | - | - | - | - | - | - | - | - | - | - | - | - | - |
| Fuentes et al. (2009) | 5 | 12 | 23 | 19 | - | - | - | - | - | - | - | - | - | - | - | - | - | - | - | - |
| Oladiji et al. (2009) | 3 | 10 | 17 | 21 | - | - | - | - | - | - | - | - | - | - | - | - | - | - | - | - |
| Snaphaan et al. (2009) | 21 | 21 | 99 | 142 | - | - | - | - | - | - | - | - | 16 | 26 | 115 | 126 | 0 | 42 | 36 | 205 |
| Sienkiewicz-Jarosz et al. (2010) | 61 | 21 | 121 | 39 | 8 | 74 | 16 | 144 | - | - | - | - | - | - | - | - | - | - | - | - |
| Nishiyama et al. (2010) | 33 | 30 | 56 | 64 | - | - | - | - | 9 | 37 | 15 | 73 | 9 | 37 | 15 | 73 | 12 | 34 | 11 | 77 |
| Bour et al. (2010) | 11 | 15 | 51 | 61 | - | - | - | - | - | - | - | - | - | - | - | - | - | - | - | - |
| Tang et al. (2010) | 20 | 58 | 16 | 62 | - | - | - | - | - | - | - | - | - | - | - | - | - | - | - | - |
| Effat et al. (2011) | - | - | - | - | - | - | - | - | 21 | 39 | 8 | 52 | 4 | 56 | 5 | 55 | - | - | - | - |
| Terroni et al. (2011) | 13 | 8 | 28 | 19 | - | - | - | - | - | - | - | - | - | - | - | - | - | - | - | - |
| Tang et al. (2011a) | - | - | - | - | - | - | - | - | 9 | 66 | 44 | 472 | - | - | - | - | 25 | 50 | 145 | 371 |
| Tennen et al. (2011) | 21 | 17 | 36 | 28 | - | - | - | - | - | - | - | - | - | - | - | - | - | - | - | - |
| Choi-Kwon et al. (2012)^*^ | 35 | 48 | 182 | 204 | - | - | - | - | 14 | 69 | 67 | 319 | - | - | - | - | - | - | - | - |
| Altieri et al. (2012) | 22 | 21 | 35 | 27 | - | - | - | - | - | - | - | - | - | - | - | - | - | - | - | - |
| Zhang et al. (2012) | 17 | 22 | 43 | 81 | - | - | - | - | 14 | 25 | 24 | 100 | - | - | - | - | - | - | - | - |
| Rajashekaran et al. (2013) | 19 | 9 | 8 | 26 | - | - | - | - | - | - | - | - | - | - | - | - | - | - | - | - |
| Gozzi et al. (2014) | 7 | 8 | 20 | 20 | - | - | - | - | 8 | 7 | 24 | 16 | - | - | - | - | - | - | - | - |
| Jiang et al. (2014) | 50 | 48 | 60 | 171 | 11 | 87 | 20 | 211 | 22 | 76 | 19 | 212 | - | - | - | - | 86 | 12 | 116 | 115 |
| Shi et al. (2014) | - | - | - | - | - | - | - | - | 33 | 187 | 76 | 771 | - | - | - | - | - | - | - | - |
| Gu et al. (2015) | - | - | - | - | - | - | - | - | 9 | 47 | 21 | 119 | - | - | - | - | 17 | 39 | 32 | 108 |
| Wichowicz et al. (2015) | 21 | 8 | 42 | 30 | - | - | - | - | 18 | 11 | 23 | 49 | - | - | - | - | 15 | 14 | 19 | 53 |
| Chen et al. (2016)^*^ | 26 | 26 | 42 | 37 | - | - | - | - | 9 | 64 | 20 | 100 | - | - | - | - | 15 | 58 | 21 | 99 |
| Wei et al. (2016)^*^ | 40 | 47 | 126 | 155 | - | - | - | - | 24 | 63 | 62 | 219 | - | - | - | - | - | - | - | - |
| Chronic stroke phase | | | | | | | | | | | | | | | | | | | | |
| House et al. (1990)^*^ | 1 | 4 | 30 | 23 | - | - | - | - | - | - | - | - | - | - | - | - | - | - | - | - |
| Sharpe et al. (1990) | 6 | 5 | 25 | 24 | - | - | - | - | - | - | - | - | - | - | - | - | - | - | - | - |
| Astrom et al. (1993)^*^ | 5 | 4 | 16 | 15 | - | - | - | - | - | - | - | - | - | - | - | - | - | - | - | - |
| Verdelho et al. (2004)^*^ | - | - | - | - | 1 | 12 | 8 | 52 | - | - | - | - | 9 | 4 | 40 | 20 | - | - | - | - |
| Provinciali et al. (2008) | 144 | 120 | 234 | 233 | - | - | - | - | - | - | - | - | - | - | - | - | - | - | - | - |
| Chatterjee et al. (2010) | 12 | 28 | 20 | 67 | - | - | - | - | - | - | - | - | 16 | 17 | 20 | 50 | - | - | - | - |
| Nidhinandana et al. (2010) | 17 | 30 | 22 | 32 | - | - | - | - | - | - | - | - | - | - | - | - | - | - | - | - |
| Srivastava et al. (2010) | 8 | 10 | 16 | 17 | 3 | 15 | 6 | 27 | - | - | - | - | - | - | - | - | - | - | - | - |
| *PSA studies* | | | | | | | | | | | | | | | | | | | | |
|  | Laterality | | | | Lesion type | | | | Frontal lesion | | | | Subcortical lesion | | | | Basal ganglia lesion | | | |
|  | PSA | | No-PSA | | PSA | | No-PSA | | PSA | | No-PSA | | PSA | | No-PSA | | PSA | | No-PSA | |
| Authors | Left | Right | Left | Right | Hem | Isch | Hem | Isch | Fr | NFr | Fr | NFr | Subc | NSubc | Subc | Nsubc | BG | nBG | BG | nBG |
| Acute stroke phase | | | | | | | | | | | | | | | | | | | | |
| Starkstein et al. (1993) | 6 | 8 | 10 | 34 | 5 | 9 | 10 | 34 | 3 | 11 | 7 | 37 | - | - | - | - | 6 | 8 | 15 | 29 |
| Piamarta et al. (2004) | 3 | 2 | 16 | 12 | - | - | - | - | - | - | - | - | - | - | - | - | - | - | - | - |
| Carota et al. (2005) | 44 | 53 | 78 | 54 | - | - | - | - | - | - | - | - | - | - | - | - | - | - | - | - |
| Glodzik-Sobanska et al. (2005) | 6 | 7 | 10 | 8 | - | - | - | - | - | - | - | - | 8 | 5 | 10 | 8 | - | - | - | - |
| Kang and Kim (2008) | 28 | 9 | 27 | 27 | - | - | - | - | - | - | - | - | - | - | - | - | - | - | - | - |
| Caeiro et al. (2012) | 7 | 20 | 24 | 17 | 13 | 23 | 9 | 49 | 4 | 5 | 3 | 8 | 9 | 9 | 17 | 11 | - | - | - | - |
| Post-acute stroke phase | | | | | | | | | | | | | | | | | | | | |
| Yamagata et al. (2004) | 7 | 9 | 4 | 9 | - | - | - | - | - | - | - | - | - | - | - | - | - | - | - | - |
| Brodaty et al. (2005) | 4 | 13 | 10 | 50 | - | - | - | - | - | - | - | - | - | - | - | - | - | - | - | - |
| Santa et al. (2008) | 8 | 6 | 19 | 34 | 3 | 11 | 29 | 24 | 5 | 41 | 30 | 123 | - | - | - | - | 21 | 25 | 61 | 92 |
| Mikami et al. (2013) | - | - | - | - | 0 | 23 | 5 | 28 | - | - | - | - | - | - | - | - | - | - | - | - |
| Tang et al. (2013a) | - | - | - | - | - | - | - | - | 2 | 18 | 22 | 143 | - | - | - | - | 4 | 16 | 22 | 143 |
| Cosin et al. (2015) | - | - | - | - | - | - | - | - | 2 | 8 | 7 | 29 | - | - | - | - | 0 | 10 | 3 | 33 |

Data represent number of patients. *BG* basal ganglia lesion*, Fr* frontal lesion*, Hem* hemorrhage, *Isch* ischemic stroke*, nBG* non basal ganglia lesion, *NFr* non-frontal lesion, *NSubc* non-subcortical lesion, *PSA* post-stroke apathy, *PSD* post-stroke depression, *Subc* subcortical lesion. ^*^Studies that provided data on more than one time point.

Imaging markers of post-stroke depression and apathy: a systematic review and meta-analysis

Elles Douven,^1^ Sebastian Köhler,^1^ Maria M.F. Rodriguez,^2^ Julie Staals,^3^ Frans R.J. Verhey,^1^ and Pauline Aalten^1*^

^1.^ Alzheimer Center Limburg, School for Mental Health and Neuroscience (MHeNS), Maastricht University Medical Center (MUMC+), Maastricht, The Netherlands.

^2.^ Complexo Universitario de Vigo, Hospital Alvaro Cunqueiro. Department of Psychiatry, Vigo, Spain.

^3.^ Department of Neurology, Cardiovascular Research Institute Maastricht (CARIM), MUMC+, Maastricht, The Netherlands.
