# Supplementary material for: Imaging Markers of Post-Stroke Depression and Apathy: a Systematic Review and Meta-Analysis
Source: Neuropsychol Rev. 2017 Aug 22;27(3):202–19. doi: 10.1007/s11065-017-9356-2 (PMC5613051; doi:10.1007/s11065-017-9356-2)
Supplement: Supplementary file 3 — (DOCX 147 kb) [file 11065_2017_9356_MOESM3_ESM.docx]

Supplementary Table 3 Newcastle-Ottowa Quality Assessment Scale

| Cohort studies (PSD) | Selection (max. 4) | | | | Comparability (max. 2) | | Outcome (max. 3) | | | Overall |
| --- | --- | --- | --- | --- | --- | --- | --- | --- | --- | --- |
| Study | S1 | S2 | S3 | S4 | C1 | C2 | O1 | O2 | O3 | Sum score |
| Robinson et al. (1984b);  Robinson et al. (1985b) | * | * | * | / | * | * | * | * | / | 7 |
| Parikh et al. (1988) | * | * | * | / | / | / | * | * | / | 5 |
| House et al. (1990) | * | * | * | / | * | * | * | * | * | 8 |
| Morris et al. (1990) | * | * | * | / | * | * | * | * | / | 7 |
| Astrom et al. (1993) | * | * | * | / | * | * | * | * | * | 8 |
| Andersen et al. (1995) | * | * | * | * | * | * | * | * | * | 9 |
| Herrmann et al. (1998) | * | / | * | / | * | * | * | * | / | 6 |
| Huwel et al. (1998) | * | * | * | / | * | * | * | * | * | 8 |
| Shimoda et al. (1999) | * | * | * | / | * | * | * | * | * | 8 |
| Singh et al. (2000) | * | * | * | * | / | / | * | * | / | 6 |
| Berg et al. (2003) | * | * | * | * | * | * | * | * | * | 9 |
| Cassidy et al. (2004) | * | / | * | / | / | * | * | * | * | 6 |
| Verdelho et al. (2004) | * | / | * | / | * | * | * | * | * | 7 |
| Paolucci et al. (2006);  Provinciali et al. (2008) | * | * | * | / | / | * | * | * | * | 7 |
| Aben et al. (2006); Leentjens et al. (2006); Bour et al. (2010) | * | * | * | / | * | * | * | * | * | 8 |
| Fuentes et al. (2009) | * | * | * | / | * | * | * | * | * | 8 |
| Iranmanesh et al. (2009) | * | / | * | * | * | * | * | * | / | 7 |
| Terroni et al. (2011) | * | * | * | * | * | * | * | * | * | 9 |
| Kim et al. (2011) | * | * | * | * | * | * | / | * | * | 8 |
| Castellanos-Pinedo et al. (2011) | * | * | * | / | * | * | * | * | / | 7 |
| Altieri et al. (2012) | * | * | * | * | * | * | * | * | * | 9 |
| Choi-Kwon et al. (2012) | * | * | * | * | / | / | * | * | * | 7 |
| Ku et al. (2013) | * | * | * | * | * | * | * | * | / | 8 |
| Zhang et al. (2013) | * | * | * | / | * | * | * | * | / | 7 |
| Shi et al. (2014) | * | * | * | * | * | * | * | * | * | 9 |
| De Ryck et al. (2014) | * | * | * | / | * | * | * | * | * | 8 |
| Gozzi et al. (2014) | * | / | * | / | / | * | * | * | * | 6 |
| Tang et al. (2014a) | * | * | * | * | * | * | / | * | / | 7 |
| Wichowicz et al. (2015) | * | * | * | / | * | * | * | * | * | 8 |
| Wei et al. (2016) | * | * | * | * | * | * | * | * | * | 9 |
| Chen et al. (2016) | * | * | * | * | * | * | * | * | * | 9 |
| Case-control studies (PSD) | Selection (max. 4) | | | | Comparability (max. 2) | | Outcome (max. 3) | | | Overall |
| Author | S1 | S2 | S3 | S4 | C1 | C2 | O1 | O2 | O3 | Sum score |
| Eastwood et al. (1989) | / | * | / | * | * | / | * | * | * | 6 |
| Dam et al. (1989) | * | * | / | * | / | / | * | * | * | 6 |
| Kase et al. (1998) | * | * | * | * | * | * | * | * | * | 9 |
| Rao et al. (2001) | * | / | / | * | * | * | * | * | * | 7 |
| Desmond et al. (2003) | * | * | * | / | * | * | * | * | * | 8 |
| Nys et al. (2005) | * | * | * | * | * | * | * | * | * | 9 |
| Glodzik-Sobanska et al. (2006) | * | * | * | * | * | * | * | * | * | 9 |
| Caeiro et al. (2006) | * | * | / | / | * | * | * | * | * | 7 |
| Brodaty et al. (2007); Withall  et al. (2011) | * | * | * | * | * | * | * | * | * | 9 |
| Xu et al. (2008) | * | * | * | / | * | / | * | * | * | 7 |
| Huang et al. (2010) | * | * | * | * | * | * | / | / | * | 7 |
| Wang et al. (2012) | * | * | * | * | * | * | * | * | * | 9 |
| Yasuno et al. (2014) | * | * | * | * | * | * | * | * | * | 9 |
| Zhang et al. (2014) | * | * | * | * | * | * | * | * | * | 9 |
| Brookes et al. (2014) | * | * | * | * | * | * | / | * | * | 8 |
| Hollocks et al. (2015) | * | * | * | * | * | * | / | * | * | 8 |
| Cross-sectional studies (PSD) | Selection (max. 4) | | | | Comparability (max. 2) | | Outcome (max. 3) | | | Overall |
| Author | S1 | S2 | S3 | S4 | C1 | C2 | O1 | | O2 | Sum score |
| Robinson et al. (1983)  Robinson et al. (1984a)  Robinson et al. (1985a) | * | * | * | / | * | * | ** | | * | 8 |
| Starkstein et al. (1988) | * | / | * | * | * | * | ** | | * | 8 |
| Starkstein et al. (1989) | * | / | * | / | * | * | ** | | * | 7 |
| Sharpe et al. (1990)  Sharpe et al. (1994) | * | * | * | / | * | * | ** | | * | 8 |
| Starkstein et al. (1991) | * | * | * | / | * | * | ** | | * | 8 |
| Stern et al. (1991) | * | / | * | * | / | / | ** | | * | 6 |
| Morris et al. (1992)  Morris et al. (1996b) | * | * | * | / | * | * | ** | | * | 8 |
| Schwartz et al. (1993) | * | * | * | / | * | * | ** | | * | 8 |
| Herrmann et al. (1993) | * | / | * | * | * | * | ** | | / | 7 |
| Herrmann et al. (1995) | * | / | * | * | * | * | ** | | * | 8 |
| Ng et al. (1996) | * | * | / | / | * | * | ** | | * | 7 |
| Gonzalez-Torrecillas et al. (1995) | * | * | * | / | * | * | ** | | * | 8 |
| Morris et al. (1996a) | * | * | * | / | * | * | ** | | * | 8 |
| Angeleri et al. (1997) | * | / | * | / | / | / | ** | | * | 5 |
| Bendsen et al. (1997) | * | / | / | * | / | * | ** | | * | 6 |
| Gainotti et al. (1997) | * | / | * | * | / | / | ** | | / | 5 |
| Pohjasvaara et al. (1998); Vataja et al. (2001); Vataja et al. (2004) | * | * | * | / | / | / | ** | | * | 6 |
| MacHale et al. (1998) | * | * | / | / | * | / | ** | | * | 6 |
| Paolucci et al. (1999) | * | * | * | * | * | * | ** | | * | 9 |
| Paradiso et al. (1999) | * | * | * | / | * | * | ** | | * | 8 |
| Gainotti et al. (1999); Gainotti  et al. (2001) | * | / | * | * | * | * | ** | | * | 8 |
| Kim et al. (2000) | * | / | * | * | * | * | / | | * | 7 |
| Hosking et al. (2000) | * | * | * | * | * | * | / | | * | 7 |
| Berg et al. (2001) | * | * | * | * | * | * | ** | | * | 9 |
| Spalletta et al. (2002)  Spalletta et al. (2005) | * | * | * | / | / | / | ** | | * | 6 |
| Piamarta et al. (2004) | * | / | * | * | * | / | ** | | / | 6 |
| Kadojić et al. (2005) | * | / | * | * | / | * | / | | / | 4 |
| Aybek et al. (2005) | * | / | * | / | / | * | ** | | / | 5 |
| Hsieh et al. (2005) | * | / | * | * | * | * | ** | | * | 8 |
| Tang et al. (2005) | * | * | * | / | / | * | ** | | * | 7 |
| Wichowicz et al. (2006) | * | / | * | * | / | / | ** | | / | 5 |
| Barker-Collo et al. (2007) | * | * | * | / | * | * | / | | * | 6 |
| Hama et al. (2007) | * | * | * | * | / | / | / | | * | 5 |
| Oladiji et al. (2009) | * | * | * | / | * | * | / | | * | 6 |
| Snaphaan et al. (2009) | * | * | * | / | * | * | / | | * | 6 |
| Chen et al. (2009) | * | * | * | * | * | * | / | | * | 7 |
| Nishiyama et al. (2010) | * | * | * | * | * | * | / | | * | 7 |
| Chau et al. (2010) | * | / | * | / | * | * | / | | * | 5 |
| Mok et al. (2010)  Fu et al. (2010) | *  * | /  / | *  * | *  * | *  * | *  * | **  / | | *  * | 8  6 |
| Sienkiewicz-Jarosz et al. (2010) | * | / | * | / | * | * | / | | * | 5 |
| Williamson et al. (2010) | * | * | * | / | * | * | / | | * | 6 |
| Nidhinandana et al. (2010) | * | * | / | * | * | * | / | | * | 6 |
| Srivastava et al. (2010) | * | * | / | / | * | * | ** | | * | 7 |
| Chatterjee et al. (2010) | * | * | * | / | * | * | ** | | * | 8 |
| Tang et al. (2010); Tang et al. (2011b); Tang et al. (2011c);  Tang et al. (2013b) | * | / | * | * | * | * | ** | | * | 8 |
| Tang et al. (2011a) | * | / | * | * | * | * | / | | * | 6 |
| Effat et al. (2011) | * | / | * | * | * | * | ** | | * | 8 |
| Tennen et al. (2011) | * | * | * | / | * | * | / | | * | 6 |
| Marasco et al. (2011) | * | * | * | / | * | * | ** | | * | 8 |
| Zhang et al. (2012) | * | * | * | * | * | * | ** | | * | 9 |
| Wongwandee et al. (2012) | * | / | * | * | * | * | ** | | * | 8 |
| Chen et al. (2013) | * | / | * | / | * | * | ** | | * | 7 |
| Rajashekaran et al. (2013) | * | * | * | / | * | * | ** | | * | 8 |
| Taylor-Pillae et al. (2013) | * | / | * | / | * | * | / | | * | 5 |
| Rashid et al. (2013) | * | / | * | / | / | * | ** | | * | 6 |
| Hosking et al. (2013) | * | * | * | * | * | * | / | | * | 7 |
| Murakami et al. (2013) | * | * | * | * | * | * | / | | * | 7 |
| Jiang et al. (2014) | * | / | * | * | * | * | ** | | * | 8 |
| Tang et al. (2014b) | * | * | * | * | * | * | / | | * | 7 |
| Stojanovic et al. (2015) | * | / | / | * | / | * | ** | | * | 6 |
| Yang et al. (2015b) | * | * | * | * | * | * | ** | | * | 9 |
| Terroni et al. (2015) | * | * | * | * | * | * | ** | | * | 9 |
| Saxena et al. (2015) | * | / | * | * | * | * | ** | | / | 7 |
| Gu et al. (2015) | * | / | * | * | * | * | ** | | * | 8 |
| Koivunen et al. (2015) | * | * | * | / | * | * | / | | * | 7 |
| Metoki et al. (2016) | * | / | * | * | * | * | ** | | * | 8 |
| Pavlovic et al. (2016) | * | * | * | * | * | * | ** | | * | 9 |
| Zhang et al. (2016) | * | / | * | * | * | * | ** | | * | 8 |
| Cohort studies (PSA) | Selection (max. 4) | | | | Comparability (max. 2) | | Outcome (max. 3) | | | Overall |
| Author | S1 | S2 | S3 | S4 | C1 | C2 | O1 | O2 | O3 | Sum score |
| Carota et al. (2005) | * | * | / | / | * | * | / | * | * | 6 |
| Castellanos-Pinedo et al. (2011) | * | * | * | / | * | * | * | * | / | 7 |
| Mikami et al. (2013) | * | / | * | * | * | * | * | * | * | 8 |
| Case-control studies (PSA) | Selection (max. 4) | | | | Comparability (max. 2) | | Outcome (max. 3) | | | Overall |
| Author | S1 | S2 | S3 | S4 | C1 | C2 | O1 | O2 | O3 | Sum score |
| Glodzik-Sobanska et al. (2005) | * | * | * | * | / | / | * | * | * | 7 |
| Brodaty et al. (2005)  Withall et al. (2011) | * | * | * | * | * | * | * | * | * | 9 |
| Caeiro et al. (2012) | * | * | / | * | / | / | * | * | * | 6 |
| Hollocks et al. (2015) | * | * | * | * | * | * | / | * | * | 8 |
| Cross-sectional studies (PSA) | Selection (max. 4) | | | | Comparability (max. 2) | | Outcome (max. 2) | | | Overall |
| Author | S1 | S2 | S3 | S4 | C1 | C2 | O1 | O2 | | Sum score |
| Starkstein et al. (1993) | * | ★ | / | / | * | * | ** | * | | 7 |
| Okada et al. (1997) | * | / | / | / | * | * | ** | * | | 6 |
| Yamagata et al. (2004) | * | / | * | * | * | * | / | * | | 6 |
| Piamarta et al. (2004) | * | / | * | * | * | / | ** | / | | 6 |
| Hama et al. (2007) | * | * | * | * | / | / | ** | * | | 7 |
| Santa et al. (2008) | * | / | * | / | * | * | ** | * | | 7 |
| Kang et al. (2008) | * | * | * | / | / | * | / | * | | 5 |
| Onoda et al. (2011) | * | / | * | * | * | * | ** | * | | 8 |
| Murakami et al. (2013) | * | * | * | * | * | * | ** | * | | 9 |
| Tang et al. (2013a) | * | * | * | * | * | * | ** | * | | 9 |
| Rochat et al. (2013) | * | / | * | / | * | / | ** | * | | 6 |
| Yang et al. (2015c) | * | / | * | * | * | / | ** | * | | 7 |
| Yang et al. (2015a) | * | / | * | * | * | * | ** | * | | 8 |
| Cosin et al. (2015) | * | * | * | * | * | * | ** | * | | 9 |
| Mihalov et al. (2016) | * | / | * | / | * | * | ** | * | | 7 |

*PSA* post-stroke apathy, *PSD* post-stroke depression

Imaging markers of post-stroke depression and apathy: a systematic review and meta-analysis

Elles Douven,^1^ Sebastian Köhler,^1^ Maria M.F. Rodriguez,^2^ Julie Staals,^3^ Frans R.J. Verhey,^1^ and Pauline Aalten^1*^

^1.^ Alzheimer Center Limburg, School for Mental Health and Neuroscience (MHeNS), Maastricht University Medical Center (MUMC+), Maastricht, The Netherlands.

^2.^ Complexo Universitario de Vigo, Hospital Alvaro Cunqueiro. Department of Psychiatry, Vigo, Spain.

^3.^ Department of Neurology, Cardiovascular Research Institute Maastricht (CARIM), MUMC+, Maastricht, The Netherlands.
