# Supplementary material for: Imaging Markers of Post-Stroke Depression and Apathy: a Systematic Review and Meta-Analysis
Source: Neuropsychol Rev. 2017 Aug 22;27(3):202–19. doi: 10.1007/s11065-017-9356-2 (PMC5613051; doi:10.1007/s11065-017-9356-2)
Supplement: Supplementary file 4 — (DOCX 169 kb) [file 11065_2017_9356_MOESM4_ESM.docx]

Supplementary Table 4 Characteristics of post-stroke depression studies

| Authors | *N*,  % Fem | Mean age | % PSD^*^ | Phase | Scale, criteria | First-ever | Design,  PT source | Imaging method | Imaging markers | | | | |  |
| --- | --- | --- | --- | --- | --- | --- | --- | --- | --- | --- | --- | --- | --- | --- |
|  |  |  |  |  |  |  |  |  | LAT | LOC | TP | LV | Other |  |
| Robinson et al. (1983)  Robinson et al. (1984a) Robinson et al. (1984b) Robinson et al. (1985a) Robinson et al. (1985b) | 103, 38.8  36, 38.9  30, 50.0  103, 43.7  61, 44.4 | 59.0  59.0  59.4  59.0  59.9 | 46.6  44.4  36.7  NS  NS | A  A  PA  A, PA  PA | HRSD, PSE, SDS  HRSD, PSE, SDS  HRSD, PSE, SDS  HRSD, PSE, SDS  HRSD, PSE, SDS | Y  Y  Y  Y  Y | CS, H/R  CS, H/R  P, H/R  CS, H/R  P, H/R | CT  CT  CT  CT  CT | X  X  X  X  X | X  X  X  X  X | - - - - - | X  X  X  X  X | DIST FP  DIST FP  DIST FP  DIST FP  DIST FP |  |
| Starkstein et al. (1988) | 26, 26.9 | 58.0 | NS | PA, C | HRSD, PSE, SDS | Y | CC, H/R | CT | - | - | - | - | ATR |  |
| Parikh et al. (1988) | 85, 40.0 | 63.0 | NS | A, PA, C | HRSD, PSE, SDS | N | P, H | CT | X | - | X | - | DIST FP |  |
| Eastwood et al. (1989) | 87, 36.8 | 63.3 | 54.0 | PA | HRSD, GDS, SDS, SADS, RDC | N | CC, R | CT | X | - | - | X | DIST FP |  |
| Dam et al. (1989) | 92, 37.0 | 52.5 | 30.4 | PA | HRSD, BDI, RDC | N | CS, H | CT | X | X | - | X | - |  |
| Starkstein et al. (1989) | 93, 38.7 | 62.2 | 30.1 | A | DSM-III, SDS, HDRS, PSE | N | CS, H | CT | - | X | X | X | DIST FP |  |
| Sharpe et al. (1990) | 60, 38.3 | 71.0 | 18.3 | C | SCID, HADS | Y | CS, C | CT | X | - | X | X | DIST FP |  |
| House et al. (1990) | 128, 54.7 | 71.2 | 10.4 | PA, C | DSM-III, PSE, BDI ≥ 13 | Y | P, C | CT | X | X | - | X | DIST FP |  |
| Starkstein et al. (1991) | 56, 41.1 | 59.0 | 46.4 | A | DSM-III, PSE, HDRS | N | P, H | CT | X | X | - | - | DIST FP |  |
| Stern and Bachman (1991) | 52, 0.0 | 65.8 | NS | C | VAMS, HRSD, DAC | Y | CS, R | CT | X | X | - | - | - |  |
| Morris et al. (1990)  Morris et al. (1992) | 99, 48.5  35, 54.0 | 70.8  68.4 | 32.3  40.0 | PA, C  PA | CIDI (DSM-III)  DSM-III, MADRS | NS  Y | P, H  CS, H | CT  CT | X  X | - - | -  X | -  X | -  DIST FP |  |
| Astrom et al. (1993) | 76, 38.8 | 72.0 | 25.0 | A, PA, C | DSM-III | N | P, H | CT | - | X | - | X | DIST FP, ATR |  |
| Schwartz et al. (1993) | 91, 0.0 | 66.0 | 39.6 | PA | DSM-III, HDRS ≥ 18 | N | CS, R | CT | X | X | - | X | ATR |  |
| Herrmann et al. (1993) | 42, 38.1 | 62.0 | NS | A | CDS, RDC | N | CS, C | CT | - | X | - | X | DIST FP |  |
| Sharpe et al. (1994) | 60, 38.3 | NS | 18.3 | C | SCID | Y | CS, C | CT | - | - | - | X | - |  |
| Andersen et al. (1995) | 285, 46.0 | 69.0 | 10.9 | PA | BDI, HDRS ≥ 13 | N | P, H | CT | X | X | - | - | - |  |
| Herrmann et al. (1995) | 47, 34.0 | 62.0 | 36.2 | PA | DSM-III-R, MADRS, CDS | Y | CS, H | CT | X | X | - | X | DIST FP, ATR |  |
| Ng et al. (1995) | 52, 44.2 | 60.0 | 55.8 | PA | DSM-III-R, HDRS | N | CS, R | CT | X | - | X | - | - |  |
| González et al. (1995) | 130, 53.8 | 67.6 | 36.9 | PA | RDC, HAM-D, MADRS, BDI | NS | CS, R | CT | X | X | - | - | - |  |
| Morris et al. (1996b) | 41, 56.1 | 70.0 | 36.6 | PA | DSM-III-R, MADRS, CIDI | Y | CS, H | CT | X | X | X | X | - |  |
| Morris et al. (1996a) | 193, 51.8 | 61.9 | 24.4 | A | CES-D ≥ 16 | Y | CS, H | CT | X | X | - | X | DIST FP |  |
| Angeleri et al. (1997) | 180, 35.0 | 63.3 | 50.0 | C | ICD-10, BDI ≥ 10 | N | CS, H | CT | X | - | - | - | - |  |
| Bendsen et al. (1997) | 128, NS | NS | 15.6 | PA | SCID, HAM-D | N | CS, R | CT | X | - | - | - | NUM, ATR |  |
| Gainotti et al. (1997) | 126, NS | NS | 39.7 | PA | DSM-III, HDRS > 18, PSDRS | N | CS, H/R | CT | X | X | - | - | - |  |
| Herrmann et al. (1998) | 150, 50.9 | 74.9 | 27.3 | PA, C | MADRS ≥ 7, SDS ≥ 50 | N | P, H | CT | X | - | - | X | - |  |
| Kase et al. (1998) | 74, 54.1 | 78.7 | 37.9 | PA | CES-D ≥ 16 | NS | CC, C | CT | X | X | - | - | - |  |
| Huwel et al. (1998) | 102, NS | 69.8 | 54.9 | A, PA | MADRS ≥ 7, SDS | Y | P, H | CT, NMR | X | X | - | - | - |  |
| Pohjasvaara et al. (1998)  Vataja et al. (2001)  Vataja et al. (2004) | 277, NS  275, 48.7  70, 50.0 | NS  70.7  70.3 | 40.1  39.6  37.1 | PA  PA  PA | PSE, MADRS ≥ 6  PSE, MADRS  DSM-III-R, ICD-10 | N  N  N | CS, H  CS, H  CS, H | MRI  MRI  MRI | X  X X | X  X  X | - - - | -  X  X | -  NUM, WMH, ATR  NUM, WMH, ATR |  |
| MacHale et al. (1998) | 55, 48.0 | 60.0 | 29.1 | PA | SADS, DSM-IV, HADS | N | CS, H | CT | X | X | - | X | DIST FP |  |
| Gainotti et al. (1999)  Gainotti et al. (2001) | 58, 41.4  64, 43.8 | 61.9  61.9 | 29.3  NS | A, PA  PA | DSM-III, HAM-D > 17, PSDRS  DSM-III, HDRS | Y  Y | CS, H/R  CS, H/R | CT, MRI  CT | X X | X - | - - | - - | - - |  |
| Paolucci et al. (1999) | 470, 51.9 | NS | 27.4 | PA | HDRS ≥ 18, VADS | Y | CS, H/R | CT, MRI | X | X | - | - | - |  |
| Paradiso et al. (1999) | 141, 40.4 | 59.2 | 38.3 | A | DSM-IV, HAM-D, PSE | N | CS, H | CT | X | X | X | X | DIST FP |  |
| Shimoda et al. (1999) | 60, 43.4 | 59.1 | 51.7 | A | DSM-IV, HDRS, PSE | Y | P, H | CT | X | X | - | X | DIST FP |  |
| Kim et al. (2000) | 148, 36.5 | 62.0 | 18.2 | PA | DSM-IV, BDI > 13 | Y | CS, H | CT, MRI | X | X | X | X | WMH |  |
| Hosking et al. (2000) | 79, 54.0 | 74.0 | 39.2 | PA | GDS > 9 | N | CS, H | CT | X | X | - | - | - |  |
| Singh et al. (2000) | 81, 38.0 | 70.0 | 35.8 | PA | SDS ≥ 50, MADRS ≥ 7 | N | P, H | CT | X | X | - | X | DIST FP |  |
| Berg et al. (2001) | 100, 32.0 | 55.2 | 27.0 | A | BDI ≥ 10, HDRS, DSM-II-R | Y | CS, H | CT, MRI | X | - | - | - | - |  |
| Rao et al. (2001) | 25, NS | NS | 56.0 | C | HDRS, GDS, DSM-IV | Y | CS/CC, C | CT | - | X | - | - | - |  |
| Spalletta et al. (2002)  Spalletta et al. (2005) | 153, 51.6  200, 58.0 | 66.3  65.6 | 57.5  56.0 | PA  PA | SCID, HDRS  SCID-P, HAM-D, BDI | Y  Y | CS, H CS, H | CT, MRI CT, MRI | X - | X - | X - | X - | - - |  |
| Desmond et al. (2003) | 421, 52.0 | 70.8 | 11.2 | PA | HDRS (SIGH-D) > 11 | N | CC, H | CT, MRI | X | X | - | - | - |  |
| Berg et al. (2003) | 89, 32.0 | 55.2 | 27.0 | A, PA, C | DSM-III, HRSD, BDI > 9 | N | P, H | NS | X | - | - | - | - |  |
| Cassidy et al. (2004) | 50, 42.0 | 51.4 | 20.0 | PA | DSM-IV, CES-D ≥16, HRSD > 7 | Y | P, R | CT, MRI | X | - | - | - | - |  |
| Verdelho et al. (2004) | 108, 52.0 | 75.0 | 42.6 | PA, C | MADRS ≥ 7, CAMDEX | N | P, H | CT, MRI | - | X | X | - | WMH, SI, ATR |  |
| Piamarta et al. (2004) | 33, 39.4 | 71.6 | 57.6 | A | PSDRS | Y | CS, H | CT, MRI | X | X | - | - | - |  |
| Aybek et al. (2005) | 254, 46.5 | 64.8 | 20.1 | A | HDRS ≥ 8, EBI ≥ 1 | Y | CS, H | CT, MRI | - | X | X | X | DIST FP |  |
| Nys et al. (2005) | 126, 53.2 | 62.3 | 52.4 | A | MADRS ≥ 8 | N | CS/CC, H | CT, MRI | X | X | X | X | WMH, SI |  |
| Hsieh and Kao (2005) | 207, 40.0 | 64.2 | 34.3 | A | HDRS > 10 | Y | CS, H | CT, MRI | X | - | - | - | - |  |
| Tang et al. (2005) | 189, 39.2 | 68.2 | 14.6 | PA | SCID | N | CS, H | CT | X | X | - | - | DIST FP, NUM |  |
| Kadojić et al. (2005) | 50, 42.0 | 65.1 | 72.0 | PA | CCEI ≥ 4.9 | N | CS, H | CT | X | - | - | - | - |  |
| Paolucci et al. (2006)  Provinciali et al. (2008) | 1064, 40.1  731, NS | 67.2  NS | 36.0  36.1 | C C | DSM-IV, BDI ≥ 10,  MADRS, VAMS | N  Y | P, H P, H | CT, MRI CT, MRI | X - | X - | X - | - - | - - |  |
| Glodzik et al. (2006) | 26, 57.7 | 63.4 | 30.8 | A, PA | DSM-IV, HDRS ≥ 13 | Y | P/CC, H | MRI, MRS | X | X | - | - | METAB |  |
| Aben et al. (2006)  Leentjens et al. (2006)  Bour et al. (2010) | 189, 47.0  165, 46.1  190, 46.8 | 68.5  68.1  68.6 | 21.7  23.0  18.8 | PA, C  PA, C  PA, C | SCID, HAM-D  SCID, HAM-D  SCID, HAM-D | Y  Y Y | P, H  P, H  P, H | CT, MRI  CT, MRI  CT, MRI | X  X  X | X  X  X | - - - | X - - | WMH, SI  WMH, SI - |  |
| Caeiro et al. (2006) | 178, 40.0 | 56.8 | 46.1 | A | DSM-IV, MADRS ≥ 7, PSDRS | N | CS/CC, H | CT, MRI | X | X | X | - | - |  |
| Wichowicz et al. (2006) | 60, 28.3 | 58.8 | 38.3 | PA | ICD-10 | N | CS, H | SPECT | X | X | - | - | rCBF |  |
| Barker-Collo (2007) | 57, 10.2 | 51.7 | 22.8 | PA | BDI-II > 19 | N | CS, H | CT | X | - | - | - | - |  |
| Hama et al. (2007) | 243, 33.3 | 65.2 | 51.9 | PA | SDS ≥ 45 | N | CS, H | CT | - | X | - | X | - |  |
| Brodaty et al. (2007)  Withall et al. (2011) | 158, 43.0  106, 39.6 | 72.1  72.1 | 12.0  10.4 | PA, C  PA | SCID  SCID | N  N | P/CC, H  CS/CC, H | CT, MRI  CT, MRI | X - | - - | - - | - - | NUM, WMH, ATR  NUM, WMH, ATR |  |
| Xu et al. (2008) | 38, 26.3 | 66.0 | All | A, PA | DSM-IV, HAM-D ≥ 7 | N | CC, H | MRS | - | - | - | - | METAB |  |
| Fuentes et al. (2009) | 59, 30.5 | 65.3 | 28.8 | PA | HDRS≥ 8 | N | P, H | CT | X | - | - | - | - |  |
| Snaphaan et al. (2009) | 283, 45.0 | 65.8 | 14.8 | PA | HADS > 8 | N | CS, H | CT, MRI | X | X | - | - | WMH, ATR |  |
| Iranmanesh et al. (2009) | 200, 55.0 | 61.2 | 32.0 | PA | DSM-IV, BDI | Y | P, H | MRI | - | X | - | - | - |  |
| Oladiji et al. (2009) | 51, 39.2 | 52.5 | 25.5 | C | DASS-21 > 9 | N | CS, H | NS | X | - | - | - | - |  |
| Chen et al. (2009) | 127, 25.2 | 64.4 | 51.2 | PA | GDS ≥ 7 | N | CS, H | MRI | - | - | X | X | NUM, WMH |  |
| Nishiyama et al. (2010) | 134, 38.8 | 67.3 | 34.3 | PA | SDS ≥ 40 | Y | CS, H | CT, MRI | - | X | - | - | - |  |
| Chau et al. (2010) | 210, 41.0 | 71.7 | 35.7 | PA | GDS 30-item > 10 | N | CS, R | CT | X | - | X | - | NUM |  |
| Mok et al. (2010) | 77, 41.6 | 75.3 | 18.2 | PA | NPI ≥ 1 symptom | N | CS, H | MRI | - | - | - | X | WMH, CMB, ATR |  |
| Fu et al. (2010) | 45, 40.0 | 74.6 | 20.0 | PA | HADS-D > 7 | N | CS, H | MRI | - | X | - | - | NUM, WMH, CMB, ATR | |
| Huang et al. (2010) | 30, 20.0 | 70.0 | All | A | DSM-IV, HAM-D | Y | CS/CC, H | MRI, MRS | - | - | - | - | METAB | |
| Sienkiewicz et al. (2010) | 242, 44.2 | 65.5 | 33.9 | PA | GDS 15-item > 5 | Y | CS, H | CT | X | X | X | - | - |  |
| Williamson et al. (2010) | 108, 49.1 | 65.0 | NS | PA | CMDI, FrSBe | N | CS, H | MRI, DTI | - | - | - | - | FA values |  |
| Nidhinandana et al. (2010) | 101, 31.7 | 60.7 | 46.5 | C | GDS (Thai) > 12 | N | CS, H | CT, MRI | X | - | - | - | - |  |
| Srivastava et al. (2010) | 51, 19.6 | 46.1 | 35.3 | C | HDRS, ICD-10 | Y | CS, R | NS | X | - | X | - | - |  |
| Chatterjee et al. (2010) | 127, 43.7 | 70.4 | 31.5 | C | DSM-IV, MADRS > 17 | N | CS, C | CT | X | X | - | X | NUM, WMH, ATR |  |
| Tang et al. (2010)  Tang et al. (2011b)  Tang et al. (2011c)  Tang et al. (2013b) | 994, 53.9  994, 53.9  591, 39.1  705, 40.1 | 66.8  66.8  66.0  66.3 | 7.8  7.8  12.7  12.1 | PA  PA  PA  PA | SCID, GDS  SCID, GDS  SCID, GDS  SCID, GDS | N N N N | CS, H CS, H CS, H CS, H | MRI MRI MRI MRI | X - - - | X - - - | - - - - | X - - - | CMB, NUM, WMH, LACI, ATR - - |  |
| Tang et al. (2011a) | 235, 39.2 | 66.3 | 35.7 | PA | GDS ≥ 7 | N | CS, H | MRI | - | X | - | X | LACI, WMH, CMB |  |
| Terroni et al. (2011) | 55, 47.1 | 51.0 | 11.8 | A, PA | SCID, HAM-D | Y | P, H | MRI | X | X | - | X | WMH |  |
| Kim et al. (2011) | 133, 46.6 | 64.7 | 47.4 | A, PA | HADS-D > 7 | N | P, H | MRI | - | X | - | - | WMH, CMB |  |
| Effat et al. (2011) | 120, 53.3 | 56.7 | NS | PA | MINI, HAM-D | N | CS, H | CT, MRI | - | X | - | - | - |  |
| Tennen et al. (2011) | 102, 49.0 | 70.8 | 37.3 | PA | CES-D ≥ 16 | N | CS, H/R | CT, MRI | X | - | - | - | - |  |
| Castellanos et al. (2011) | 89, 48.3 | X | 42.7 | PA | HAM-D > 7, NPI | N | P, H | CT, MRI | X | X | - | - | - |  |
| Marasco et al. (2011) | 54, 27.8 | 65.3 | 40.7 | A | PSDRS ≥ 9 | Y | CS, H | CT | X | X | - | - | - |  |
| Altieri et al. (2012) | 105, 34.3 | 64.4 | 21.9 | PA | BDI-SF, SCID | N | P, H | NS | X | X | - | - | WMH, ATR |  |
| Zhang et al. (2012) | 163, NS | X | 23.9 | PA | DSM-IV, NPI, WHO-CIDI | N | CS, H | MRI | X | X | - | X | WMH |  |
| Choi-Kwon et al. (2012) | 508, 38.8 | 62.1 | 13.7 | A, PA | DSM-IV, BDI > 13 | N | P, H | MRI | X | X | - | - | WMC, CMB |  |
| Wang et al. (2012) | 51, 41.2 | 61.1 | 21.6 | PA | HAM-D ≥ 14, SCID | Y | CS/CC, H | MRI, MRS | - | - | - | - | METAB |  |
| Wongwandee et al. (2012) | 39, 33.3 | 59.7 | 28.2 | A | HDRS > 7, DSM-III-R | Y | CS, H | CT, MRI | X | X | - | - | - |  |
| Chen et al. (2013) | 102, 32.2 | 72.6 | 30.4 | A | HDRS > 10 | N | CS, H | CT, MRI | X | - | - | - | - |  |
| Ku et al. (2013) | 26, 26.9 | 60.8 | 11.5 | A | MINI, HADS | N | P, H | MRI | X | X | - | X | - |  |
| Zhang et al. (2013) | 91, 36.3 | 60.0 | 27.5 | A, PA | HAM-D ≥ 7, WHO-CIDI | N | P, H | CT, MRI | X | - | X | - | - |  |
| Rajashekaran et al. (2013) | 62, 21.0 | 57.9 | 45.2 | PA | MINI, MADRS, BDI | Y | CS, H | CT, MRI | X | X | - | - | - |  |
| Taylor-Piliae et al. (2013) | 100, 46.0 | 70.0 | 35.0 | C | CES-D ≥ 16 | N | CS, C | NS | X | - | X | - | - |  |
| Rashid et al. (2013) | 60, 28.3 | 67.4 | NS | C | PSDRS | N | CS, H | NS | X | - | - | - | - |  |
| Hosking and Marsh (2013) | 67, 52.0 | 74.0 | 32.8 | C | GDS > 9 | N | CS, H | CT | X | X | - | - | - |  |
| Murakami et al. (2013) | 149, 34.9 | 66.8 | 44.3 | PA | HADS ≥ 8 | N | CS, H | MRI | - | X | - | - | - |  |
| Shi et al. (2014) | 1067, 35.2 | 61.5 | 28.4 | A, PA, C | DSM-IV, HRSD | Y | P, H | CT, MRI | - | X | - | - | - |  |
| Yasuno et al. (2014) | 29, 20.7 | 68.7 | NS | PA | HAM-D, SDS | N | P, H | MRI, DTI | - | - | - | - | - |  |
| De Ryck et al. (2014) | 94, 42.8 | 70.1 | 24.5 | PA, C | CSD ≥ 8, MADRS | N | P, H | CT, MRI | X | - | X | - | - |  |
| Jiang et al. (2014) | 392, 30.4 | 67.2 | 25.0 | PA | MDD: DSM-IV, MADRS ≥ 8 | N | CS, H | MRI | X | X | X | - | NUM |  |
| Gozzi et al. (2014) | 55, 43.6 | 62.9 | 27.3 | PA | HADS ≥ 11, MINI | Y | P, H | MRI | X | X | - | X | - |  |
| Zhang et al. (2014) | 50, 22.0 | 58.2 | 52.0 | A | DSM-IV, HDRS > 7 | Y | CC, H | MRI, fMRI | - | - | - | - | FC in ROIs |  |
| Brookes et al. (2014) | 101, 31.0 | 70.1 | 29.7 | C | GDS > 9 | N | CS, H | MRI, DTI | - | - | - | - | Median FA |  |
| Tang et al. (2014b)  Tang et al. (2014a) | 229, 29.3  135, 51.1 | 68.8  65.7 | 32.8  NS | PA PA, C | GDS ≥ 7  GDS ≥ 7 | N N | CS, H  P, H | MRI MRI | - - | X - | - - | X - | CMB, NUM, WMH - |  |
| Stojanovic et al. (2015) | 118, 50.0 | X | 11.0 | PA | HRSD > 7 | N | CS, H | CAT | - | X | - | X | - |  |
| Yang et al. (2015) | 116, 34.5 | 67.8 | 12.1 | A | DSM-IV, HAM-D ≥ 20 | N | CS, H | MRI, DTI | - | X | - | - | FA maps |  |
| Terroni et al. (2015) | 36, 41.7 | 51.5 | 19.4 | A | SCID | Y | CS, H | MRI | X | X | - | X | - |  |
| Hollocks et al. (2015) | 118, 34.7 | 68.9 | NS | PA | GDS (24-item) | Y | CS, H | MRI, DTI | - | - | - | - | FA, WM networks |  |
| Saxena and Suman (2015) | 107, 43.9 | 59.1 | 57.0 | A | MADRS > 6 | N | CS, H | CT, MRI | X | X | X | X | - |  |
| Gu et al. (2015) | 196, 33.7 | 61.2 | 28.6 | PA | DSM-IV, HAM-D | N | CS, H | CT, MRI | - | X | - | - | - |  |
| Wichowicz et al. (2015) | 116, 36.2 | 61.4 | 22.9 | A, PA, C | ICD-10, HRSD | Y | P, H | CT, MRI | X | X | - | - | - |  |
| Koivunen et al. (2015) | 130, 51.5 | 41.5 | 23.1 | C | BDI > 13, HADS | Y | CS, H | CT, MRI | - | X | - | X | NUM |  |
| Wei et al. (2016) | 368, 29.1 | 61.5 | 19.3 | A, PA | DSM-IV, BDI > 13 | N | P, H | CT, MRI | X | X | - | - | WMH |  |
| Chen et al. (2016) | 207, 27.5 | 60.8 | 41.1 | A, PA | HDRS ≥ 8 | N | P, H | MRI | X | X | - | X | NUM, WMH, ATR |  |
| Metoki et al. (2016) | 421, 37.3 | 72.1 | 16.9 | A | JSS-D ≥ 2.4 | NS | CS, H | MRI | X | X | - | - | - |  |
| Pavlovic et al. (2016) | 294, 46.3 | 62.3 | 39.8 | C | DSM-IV, HDRS | Y | CS, H | MRI | - | - | - | - | LACI, WMH |  |
| Zhang et al. (2016) | 251, 27.9 | 66.0 | 17.9 | A | HAM-D ≥ 7 | N | CS, H | CT, MRI | X | X | - | - | - |  |

*A* acute, *ATR* atrophy, *C* chronic (phase), *BDI* Beck Depression Inventory, *BDI-II* Beck Depression Inventory Second Edition, *BDI-SF* Beck Depression Inventory Short Form, *C* community (patient source), *CAMDEX* Cambridge Mental Disorders of the Elderly Examination, *CC* case-control, *CCEI* Crown-Crips Experiential Index, *CDS, CSD* Cornell Scale for Depression, *CES-D* Center for Epidemiologic Studies Depression Scale, *CIDI* Composite International Diagnostic Interview, *CMB* cerebral microbleeds, *CMDI* Chicago Multiscale Depression Inventory, *CS* cross-sectional, *CT* computed tomography, *DAC* Depression Adjective Checklist, *DASS-21*, Depression Anxiety Stress Scale 21-item version, *DIST FP* distance to frontal pole, *DSM-III* Diagnostic and Statistical Manual of Mental Disorders Third Edition, *DSM-III-R* Diagnostic and Statistical Manual of Mental Disorders Third Edition revised, *DSM-IV* Diagnostic and Statistical Manual of Mental Disorders Fourth Edition, *DTI* diffusion tensor imaging, *EBI* Emotional Behavior Index, *FA* fractional anisotropy, *Fem* female, *fMRI* functional magnetic resonance imaging, *FrSBe* Frontal Systems Behavior Scale, *GDS* Geriatric Depression Scale, *H* hospital, *HADS* Hospital Anxiety and Depression Scale, *HRSD, HDRS, HAM-D* Hamilton Rating Scale for depression, *ICD-10* International Statistical Classification of Diseases and Related Health Problems 10th Revision, *JSS-D* Japan Stroke Scale Depression Scale, *LACI* lacunar infarcts, *LAT* laterality, *LOC* location, *LV* lesion volume, *MADRS* Montgomery Åsberg Depression Rating Scale, *METAB* metabolism, *MINI* Mini International Neuropsychiatric Interview, *MRI* magnetic resonance imaging, *MRS* proton magnetic resonance spectroscopy, *N* number of participants, *N* no, *NMR* nuclear magnetic resonance, *NPI* Neuropsychiatric Inventory, *NS* not specified, *NUM* number of lesions, *P* prospective, *PA* post-acute, *PSD* post-stroke depression, *PSDRS* Post-Stroke Depression Rating Scale, *PSE* Present State Examination, *PT* patient, *R* rehabilitation center, *rCBF* regional cerebral blood flow, *RDC* Research Diagnostic Criteria, *ROIs* regions of interest, *SADS* Schedule for Affective Disorders and Schizophrenia, *SCID* Structured Clinical Interview for DSM Disorders, *SCID-P* Structured Clinical Interview for DSM Disorders Patient Edition, *SDS* Zung Self-rating Depression Scale, *SI* silent infarcts, *SIGH-D* Structured Interview Guide for the HDRS, *SPECT* single-photon emission computed tomography, *TP* type of stroke, *VAMS* Visual Analogue Mood Scale, *WHO-CIDI* World Health Organization Composite International Diagnostic Interview, *WM* white matter, *WMC* white matter changes, *WMH* white matter hyperintensities, *Y* yes.^*^Percentage of participants with PSD at baseline measurement

Imaging markers of post-stroke depression and apathy: a systematic review and meta-analysis

Elles Douven,^1^ Sebastian Köhler,^1^ Maria M.F. Rodriguez,^2^ Julie Staals,^3^ Frans R.J. Verhey,^1^ and Pauline Aalten^1*^

^1.^ Alzheimer Center Limburg, School for Mental Health and Neuroscience (MHeNS), Maastricht University Medical Center (MUMC+), Maastricht, The Netherlands.

^2.^ Complexo Universitario de Vigo, Hospital Alvaro Cunqueiro. Department of Psychiatry, Vigo, Spain.

^3.^ Department of Neurology, Cardiovascular Research Institute Maastricht (CARIM), MUMC+, Maastricht, The Netherlands.
