# Supplementary material for: Imaging Markers of Post-Stroke Depression and Apathy: a Systematic Review and Meta-Analysis
Source: Neuropsychol Rev. 2017 Aug 22;27(3):202–19. doi: 10.1007/s11065-017-9356-2 (PMC5613051; doi:10.1007/s11065-017-9356-2)
Supplement: Supplementary file 5 — (DOCX 135 kb) [file 11065_2017_9356_MOESM5_ESM.docx]

| Study | Year | *N*, % Fem | Mean age | % PSA^*^ | Phase | Scale | First-ever | Design,  PT source | Imaging method | Imaging markers | | | | |
| --- | --- | --- | --- | --- | --- | --- | --- | --- | --- | --- | --- | --- | --- | --- |
|  |  |  |  |  |  |  |  |  |  | LAT | LOC | TP | LV | Other |
| Starkstein et al. (1993) | 1993 | 80, 46.3 | 59.5 | 22.5 | A | AS ≥ 14 | Y | CS, H | CT | X | X | X | X | - |
| Okada et al. (1997) | 1997 | 40, 42.5 | 71.4 | 50.0 | C | AS ≥ 16 | N | CS, H | CT, MRI | - | X | - | - | rCBF |
| Yamagata et al. (2004) | 2004 | 29, 72.4 | 71.7 | 55.2 | PA | AS ≥ 16 | N | CS, H | MRI | X | X | - | X | NUM |
| Piamarta et al. (2004) | 2004 | 33, 39.4 | 71.6 | 15.2 | A | PSDRS expanded > 4 | Y | CS, H | CT, MRI | X | X | - | - | - |
| Carota et al. (2005) | 2005 | 273, 46.9 | 64.4 | 47.6 | A | EBIF ≥ 0.5 | Y | P, H | CT, MRI | X | X | - | - | - |
| Glodzik et al. (2005) | 2005 | 31, 48.4 | 62.9 | 41.9 | A | AS > 16 | Y | CC, H | CT, MRI, MRS | X | X | - | - | METAB |
| Brodaty et al. (2005)  Withall et al. (2011) | 2005  2011 | 135, 39.3  106, 39.6 | 72.2  72.1 | 26.7  25.5 | PA  PA | AES ≥ 37  AES ≥ 37 | Y Y | CC, H  P, H | CT, MRI CT, MRI | X - | - - | - - | X - | NUM, ATR, WMH ATR, WMH |
| Hama et al. (2007) | 2007 | 243, 33.3 | 65.2 | 40.3 | PA | AS ≥ 16 | N | CS, H | CT | - | X | - | X | - |
| Santa et al. (2008) | 2008 | 67, 43.3 | 65.4 | 20.9 | PA | AS > 15 | Y | CS, R | MRI | X | X | X | - | - |
| Kang and Kim (2008) | 2008 | 100, 42.0 | 65.1 | 43.0 | A | Clinical observation | N | CS, H | MRI, MRA | X | X | - | - | - |
| Onoda et al. (2011) | 2011 | 102, 44.1 | 73.0 | 36.3 | A, PA | AS ≥ 16 | N | CS, H | MRI, SPECT | - | X | - | - | rCBF |
| Castellanos et al. (2011) | 2011 | 89, 48.3 | 70.0 | 13.5 | PA | NPI | N | P, H | CT, MRI | X | X | - | - | - |
| Caeiro et al. (2012) | 2012 | 94, 35.1 | 55.7 | 38.3 | A | AES-10 ≥ 18 | N | CC, H | CT, MRI | X | X | X | - | - |
| Murakami et al. (2013) | 2013 | 149, 34.9 | 66.8 | 44.3 | PA | AS ≥ 16 | N | CS, H | MRI | - | X | - | - | - |
| Mikami et al. (2013) | 2013 | 56, 35.7 | 63.9 | 41.1 | PA, C | AS, criteria Robert | N | P, H/C | NS | - | - | X | - | - |
| Tang et al. (2013a) | 2013 | 185, 37.3 | 65.2 | 10.8 | PA | AES ≥ 37 | N | CS, H | MRI | - | X | - | X | NUM, WMH |
| Rochat et al. (2013) | 2013 | 55, NS | 56.4 | NS | PA, C | AI | Y | CS, H | CT, MRI | - | X | - | X | - |
| Yang et al. (2015c) | 2015 | 54, 35.2 | 67.4 | 57.4 | PA | AES, criteria Robert | N | CS, H | MRI, DTI | - | X | - | - | FA maps |
| Yang et al. (2015a) | 2015 | 88, 27.3 | 68.2 | 38.6 | A, PA | AES, criteria Robert | N | CS, H | MRI, DTI | - | - | - | X | WM integrity/networks |
| Cosin et al. (2015) | 2015 | 46, 17.4 | 58.0 | 21.7 | PA | AI > 2, LARS > 21 | N | CS, H | MRI | - | X | - | - | LACI, WMH, CMB |
| Hollocks et al. (2015) | 2015 | 118, 34.7 | 68.9 | NS | PA | GDS (6-items) | Y | CS, H | MRI, DTI | - | - | - | - | FA,WM networks |
| Mihalov et al. (2016) | 2016 | 47, 36.2 | 67.7 | 23.4 | PA | AS ≥ 14 | N | CS, H | CT, MRI | - | - | - | - | ATR |

Supplementary Table 5 Characteristics of post-stroke apathy studies

*A* acute, *AES* Apathy Evaluation Scale, *AES-10* Apathy Evaluation Scale 10-item, *AS* Apathy Scale, *ATR* atrophy, *C* chronic (phase), *C* community (patient source), *CC* case-control, *CMB* cerebral microbleeds, *CS* cross-sectional, *CT* computed tomography, *DTI* diffusion tensor imaging, *EBIF* Emotion Behavior Index Form, *FA* fractional anisotropy, *Fem* female, *H* hospital, *LACI* lacunar infarcts, *LARS* Lille Apathy Rating Scale, *LAT* laterality, *LOC* location, *LV* lesion volume, *METAB* metabolism, *MRA* magnetic resonance angiography, *MRI* magnetic resonance imaging, *MRS*=proton magnetic resonance spectroscopy, *N* number of participants, *N* no, *NPI* Neuropsychiatric Inventory, *NS* not specified, *NUM* number of lesions, *P* prospective, *PA* post-acute, *PSA* post-stroke apathy, *PSDRS* Post-Stroke Depression Rating Scale, *PT* patient, *R* rehabilitation center, *rCBF* regional cerebral blood flow, *SPECT* single-photon emission computed tomography, *TP* type of stroke,*WM* white matter, *WMH* white matter hyperintensities. *Y* yes.^*^Percentage of participants with PSA at baseline measurement.

Imaging markers of post-stroke depression and apathy: a systematic review and meta-analysis

Elles Douven,^1^ Sebastian Köhler,^1^ Maria M.F. Rodriguez,^2^ Julie Staals,^3^ Frans R.J. Verhey,^1^ and Pauline Aalten^1*^

^1.^ Alzheimer Center Limburg, School for Mental Health and Neuroscience (MHeNS), Maastricht University Medical Center (MUMC+), Maastricht, The Netherlands.

^2.^ Complexo Universitario de Vigo, Hospital Alvaro Cunqueiro. Department of Psychiatry, Vigo, Spain.

^3.^ Department of Neurology, Cardiovascular Research Institute Maastricht (CARIM), MUMC+, Maastricht, The Netherlands.
