# Supplementary material for: Imaging Markers of Post-Stroke Depression and Apathy: a Systematic Review and Meta-Analysis
Source: Neuropsychol Rev. 2017 Aug 22;27(3):202–19. doi: 10.1007/s11065-017-9356-2 (PMC5613051; doi:10.1007/s11065-017-9356-2)
Supplement: Supplementary file 6 — (DOCX 347 kb) [file 11065_2017_9356_MOESM6_ESM.docx]

Supplementary Figures

**Fig 1*.*** Funnel plots to visualize potential (publication) bias in post-stroke depression studies

**Fig 2.** Funnel plots to visualize potential (publication) bias in post-stroke apathy studies

Imaging markers of post-stroke depression and apathy: a systematic review and meta-analysis

Elles Douven,^1^ Sebastian Köhler,^1^ Maria M.F. Rodriguez,^2^ Julie Staals,^3^ Frans R.J. Verhey,^1^ and Pauline Aalten^1*^

^1.^ Alzheimer Center Limburg, School for Mental Health and Neuroscience (MHeNS), Maastricht University Medical Center (MUMC+), Maastricht, The Netherlands.

^2.^ Complexo Universitario de Vigo, Hospital Alvaro Cunqueiro. Department of Psychiatry, Vigo, Spain.

^3.^ Department of Neurology, Cardiovascular Research Institute Maastricht (CARIM), MUMC+, Maastricht, The Netherlands.
